# Exploring DIX-DIX Homo- and Hetero-Oligomers in Wnt Signaling with AlphaFold2

**DOI:** 10.3390/cells13191646

**Published:** 2024-10-03

**Authors:** Zehua Wen, Lei Wang, Shi-Wei Liu, Hua-Jun Shawn Fan, Jong-Won Song, Ho-Jin Lee

**Affiliations:** 1 College of Chemical Engineering, Sichuan University of Science and Engineering, Zigong 64300, China; zehuawen5733@163.com (Z.W.); 322086001105@stu.suse.edu.cn (L.W.); liushiwei1995@163.com (S.-W.L.); 2 Department of Chemistry Education, Daegu University, Daegudae-ro 201, Gyeongsan-si 38453, Gyeongsangbuk-do, Republic of Korea; sjoshua@daegu.ac.kr; 3 Division of Natural & Mathematical Sciences, LeMoyne-Owen College, Memphis, TN 38126, USA

**Keywords:** AlphaFold2, PRODIGY, DIX, binding affinity, Wnt signaling

## Abstract

Wnt signaling is involved in embryo development and cancer. The binding between the DIX domains of Axin1/2, Dishevelled1/2/3, and Coiled-coil-DIX1 is essential for Wnt/β-catenin signaling. Structural and biological studies have revealed that DIX domains are polymerized through head-to-tail interface interactions, which are indispensable for activating β-catenin Wnt signaling. Although different isoforms of Dvl and Axin proteins display both redundant and specific functions in Wnt signaling, the specificity of DIX-mediated interactions remains unclear due to technical challenges. Using AlphaFold2(AF2), we predict the structures of 6 homodimers and 22 heterodimers of DIX domains without templates and compare them with the reported X-ray complex structures. PRODIGY is used to calculate the binding affinities of these DIX complexes. Our results show that the Axin2 DIX homodimer has a stronger binding affinity than the Axin1 DIX homodimer. Among Dishevelled (Dvl) proteins, the binding affinity of the Dvl1 DIX homodimer is stronger than that of Dvl2 and Dvl3. The Coiled-coil-DIX1(Ccd1) DIX homodimer shows weaker binding than the Axin1 DIX homodimer. Generally, heterodimer interactions tend to be stronger than those of homodimers. Our findings provide insights into the mechanism of the Wnt signaling pathway and highlight the potential of AF2 and PRODIGY for studying protein–protein interactions in signaling pathways.

## 1. Introduction

Wnt signaling pathways are essential in embryo development and cancer [1,2,3,4,5,6,7]. In the absence of Wnt signaling, β-catenin degradation mediated by a destruction complex containing Axin, adenomatous polyposis coli (APC), glycogen synthase kinases-3β (GSK-3β), and casein kinase I (CK1) is observed [8]. The canonical Wnt signaling is initiated and controlled by many protein–protein interactions, such as Wnt-Frizzled-LRP5/6-Dvl signalosomes [9,10,11,12,13,14]. Characterizing these protein–protein interactions is essential to understand the mechanism of action of the canonical Wnt pathway. The activation of canonical Wnt signaling stabilizes a key effector, β-catenin. The accumulated β-catenin proteins translocate to the nucleus and bind to the T cell factor/lymphoid enhancer-binding factor (TCF/LEF) transcription factors to transcribe Wnt target genes.

**Figure 1:**
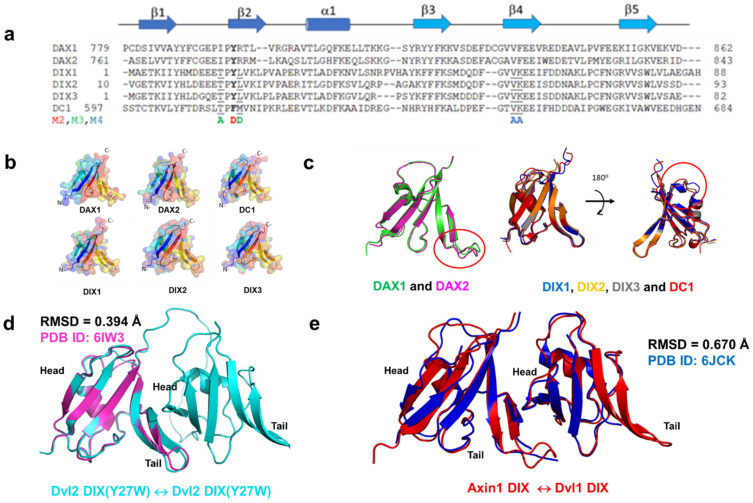
Three proteins, human Axin, Dishevelled (Dvl), and Coiled-coil-DIX1 (Ccd1), have a DIX domain. (**a**) Sequence alignment of the DIX domain of the three proteins, their paralogs, and their mutants (M2, M3, and M4). The secondary structure of the DIX domain is shown (blue, head; sky blue, tail). (**b**) AlphaFold2(AF2) successfully predicted the monomer structures of all six DIX domains with high accuracy compared to the corresponding X-ray structures. The surface and cartoon structures of DIX domains are presented. (**c**) The overlap of the predicted structures of DAX1 and DAX2; those of DIX1/2/3 and DC1 show similar folds overall. (**d**) The AF2-powered ColabFold-predicted homodimer of Dvl2 DIX(Y27W)-Dvl2 DIX(Y27W) and (**e**) the AF2-powered ColabFold-predicted heterodimer Axin1 DIX-Dvl1 DIX are shown as cartoons. The calculated RMSD values of the two models are shown, indicating the excellent agreement between the AlphaFold2 prediction and the experimental one. No template was used during AlphaFold2 prediction.

Three DIX proteins, Axin, Dishevelled (Dvl), and Coiled-coil-DIX1(Ccd1), are central components of the canonical Wnt signal transduction machinery (Figure 1a) [15,16,17,18,19,20]. For Dvl proteins, the DIX domain is located at the N terminus. For Axin1/2 and Ccd1 proteins, the DIX domain is located at the C-terminus. Through their DIX domains, these proteins can form dynamic homo- or heteropolymers in vitro and in vivo, resulting in the autoinhibition or the activation of downstream Wnt signaling [10,11,12,16,20,21,22,23]. However, the precise mechanism of DIX-mediated homomeric or heteromeric polymerization remains unknown because of their paralogs (Axin1/2, Dvl 1/2/3, and Ccd1), and their polymerization depends on the intracellular concentration. The roles of individual human Axin and Dvl paralogs in Wnt signaling interest researchers, showing their redundant and specific functions [24,25].

Axin1 serves as a scaffold for the β-catenin destruction complex [26]. The related protein Axin2/Conductin/Axil is assumed to perform a similar function [27], while Jho et al. reported that Axin2 is a negative regulator of the signaling [28]. Axin1 is expressed ubiquitously, and Axin2 is expressed in tissue- and developmental-stage-specific canonical Wnt signaling [29]. Dvl proteins are critical intracellular signaling molecules found in the cytoplasm and in the nucleus [30]. Three Dvl paralogs are present in humans [24,29]. Researchers reported that the expression patterns and developmental functions of the Dvl paralogs are only partially redundant [24,25,29]. Without Wnt signaling, Dvl is inactive due to autoinhibition [31,32]. The researchers found that the conformational change in Dvl is significant to the initiation and distinguishing of Wnt signaling [31,32,33,34]. Several studies demonstrated that Dvl DIX polymerization is critical during the initiation of canonical Wnt signaling [10,19,35]. Dvl polymers can interact directly with Axin1/2 DIX domains upon the activation of Wnt signaling [22]. Similar to Axin1/2, Ccd1 plays a role in converting latent polymeric Dvl to biologically active oligomers [18,19,20,36]. An immunoprecipitation assay showed that the Ccd1 DIX interacts with the Dvl DIX and Axin DIX domains [19,20]. Liu et al. showed the heteromeric interaction between the head interface of Dvl2-DIX and the tail interface of Ccd1-DIX, but not vice versa [19].

DIX domains are ubiquitin-like fold structures comprising five strands and one α-helix (Figure 1) [20,36,37,38]. In addition, the DIX domain’s homo- and heteropolymers adopt a head-to-tail interface interaction (Figure 1d,e). Based on the structural information, researchers could identify the essential residues that allow the dimerization or oligomerization of DIX domains to occur [16,19,20,37,38,39,40,41]. Point-directed mutagenesis led to the discovery that the loop β1-β2 regions of DIX domain-containing proteins are essential to forming the head-to-tail structure (Figure 1) [20,36]. Despite the complex structure information (SI Table S1), the binding affinities and specificity of DIX-containing proteins still need to be improved due to the technical challenges of DIX proteins. Since DIX domains can form dimers or oligomers despite low concentrations, purifying the monomer form of the DIX domain is a considerable challenge. Thus, many studies have used mutants to obtain the binding affinities of the DIX domains of homodimers and heterodimers [19,20]. However, DIX mutants can still form an oligomer or dimer at a low concentration, and the mutant’s protein stability is not certain [12].

We employed AlphaFold2(AF2) and PRODIGY (PROtein binDIng enerGY prediction) to examine the binding and specificity of DIX domain-mediated interactions. AF2 is a superb tool for highly accurate protein structure prediction [42,43,44]. AF2 stimulates applications such as ColabFold and AlphaFold-multimers to improve protein–protein interaction prediction [45,46,47]. In this study, we evaluated the performance of the AF2 prediction of the DIX dimerization as benchmarks by comparison of the X-ray structures. The PRODIGY was used to predict binding affinity in the DIX-mediated interactions from their predicted 3D structures [48,49]. PRODIGY calculates the number of interfacial contacts between proteins and uses this to estimate the binding affinity [48,49]. Our results support that AF2 is an easy-to-use and fast tool that accurately generates 3D structures of the DIX domain’s complexes. PRODIGY can be used to explore the binding specificity of DIX-mediated interactions. Our computational results suggest that DIX-mediated interactions among three proteins and their paralogs may explain a distinct mechanism of action in the Wnt signaling, although other factors need to be considered.

## 2. Methods

ColabFold (https://github.com/sokrypton/ColabFold, accessed on 22 August 2022), for use as a Jupyter Notebook inside Google Colaboratory, was employed to generate 32 complex structures of human wild-type DIX domains, including 4 mutants [42,45,46]. No template was used during the calculations. SI Table S2 lists the input sequence information. We used two methods to generate the complex structures (SI Figure S1). The first method runs AlphaFold2 with a 200 residue gap in the residue index between chains, as implemented in ColabFold [46]. The binding mode used was 1:1. The second method used here is inserting the extra amino acid residues between two target proteins. Eighteen residues, 6x GGS amino acid residues, were used to link two proteins. The predicted Dvl2 DIX-M2(Y27W)-(GGS)_6_-Dvl2 DIX-M2(Y27W) complex structure (PDB ID:6IW3) and Axin1 DIX-(GGS)_6_-Dvl2 DIX complex structure (PDB ID:6JCK) were used as benchmarks. We used the nomenclature of mutants M2, M3, and M4 based on Fielder et al.’s work [11]. The mutation’s location is outside the complex interfaces of DIX domains, which should not affect the protein–protein interaction to form homodimers or heterodimers. The MMSeqs2 (UniRef and Environmental) and MSA mode (unpaired + paired) were selected during the calculation. AF2 generated five models for each complex. The value of pLDDT was used to rank the predicted models with different confidence levels (SI Figure S6). The top-ranked complex model structure of the DIX homodimer and heterodimer was selected for further analysis. We removed the 6xGGS linker in the predicted complex structure using AF2 and optimized it with the amber force field implemented in HyperChem software [50]. Then, the free Gibbs free energy (△G in kcal/mol) and binding affinity (K_D_ value, μM) of these 32 optimized complexes (SI Table S3) at 25 °C were calculated using the PRODIGY web server, which requires a complex three-dimensional structure as input. Using the PRODIGY, we also investigated the residues participating in the DIX-mediated interactions [48]. For comparison, the SWISS-MODEL (https://swissmodel.expasy.org/, accessed on 20 October 2022) and GalaxyWEB [51] (were used to predict the structures of the homodimers and heterodimers of DIX domains (SI Figure S7. https://galaxy.seoklab.org/, accessed on 22 October 2022). The HawkDock was also used to calculate the binding energies of the DIX domains using the Molecular mechanics generalized Born surface area (MM/GBSA) method [52] (SI Table S4. http://cadd.zju.edu.cn/hawkdock/, accessed on 11 October 2022).

## 3. Results and Discussion

### 3.1. Evaluation of AF2-Based Prediction with a Comparison of the Reported Complex Structures of the DIX Domains

To investigate the accuracy of AF2, we generated the 3D structures of the monomer form of the DIX domains from the sequence of three proteins and their paralogs (Axin1/2, Dvl1/2/3, and Ccd1) without any template using ColabFold [46] (Figure 1a,b). AF2 predicted the 3D structures of the DIX domains with high accuracy. The predicted monomer DIX structures are readily superimposable to the previously reported structure of DIX proteins, with an overall root mean square deviation (RMSD) of less than 0.5 Å. Our results also support the outperformance of AF2 in predicting the 3D structure of monomer DIX domains [53].

We then generated the homodimer of human Dvl2 DIX-M2(Y27W) mutant and heterodimer of wild-type human Axin1 DIX (DAX1) and human Dvl1 DIX (DIX1) to evaluate the performance of AF2 by comparison of the reported corresponding X-ray structures (Figure 1d,e; SI Table S1) [37,40]. Both complexes of DIX domains showed the head-to-tail interaction as reported (Figure 1d,e and SI Figure S2). The orientation of the side chain of the amino acid residues in the predicted DIX homodimer and heterodimer is also quite similar to the experimental results (SI Figure S2). The backbone RMSD between the predicted and X-ray structures is also less than 0.5 Å, supporting that AF2 can accurately predict DIX-DIX complex structures [44,54].

We further evaluated the AF2 prediction complex using the human DIX mutants, DIX2-M4(Y27D)-(GGS)_6_-DAX1-M2(V800A/F801A) and DAX1-M2-(GGS)_6_-DIX2-M4 mutants (SI Figure S2). DIX2-M4(Y27D) is a mutant in the head interface. DAX2-M2(V800A/F801A) is a mutant in the tail interface [11]. Since the mutations are located outside the binding interfaces, we expected that DIX2-M4-(GGS)_6_-DAX1-M2 would form the complex through the tail interface of DIX2-M4 with the head interface of DAX1-M2 (SI Figure S2a). Indeed, AF2 predicted the complex structure of DIX2-M4(Y27D)-(GGS)_6_-DAX1-M2(V800A/F801A) [46]. As a negative control, the complex structure of DAX1-M2(V800A/F801A)-(GGS)_6_-DIX2-M4(Y27D) was also generated to see whether it might predict the head-to-tail interaction. Interestingly, the expected complex of DAX1-M2-(GGS)_6_-DIX2-M4 showed diverse structures. The two structures are the same as that of DIX2-M4(Y27D)-(GGS)_6_-DAX1-M2(V800A/F801A). One predicted complex showed no interaction between the two DIX mutants (SI Figure S2a) [16]. The results suggest that AF2 is an excellent method for predicting the 3D structures of the homodimers and heterodimers of DIX-containing proteins.

**Figure 2:**
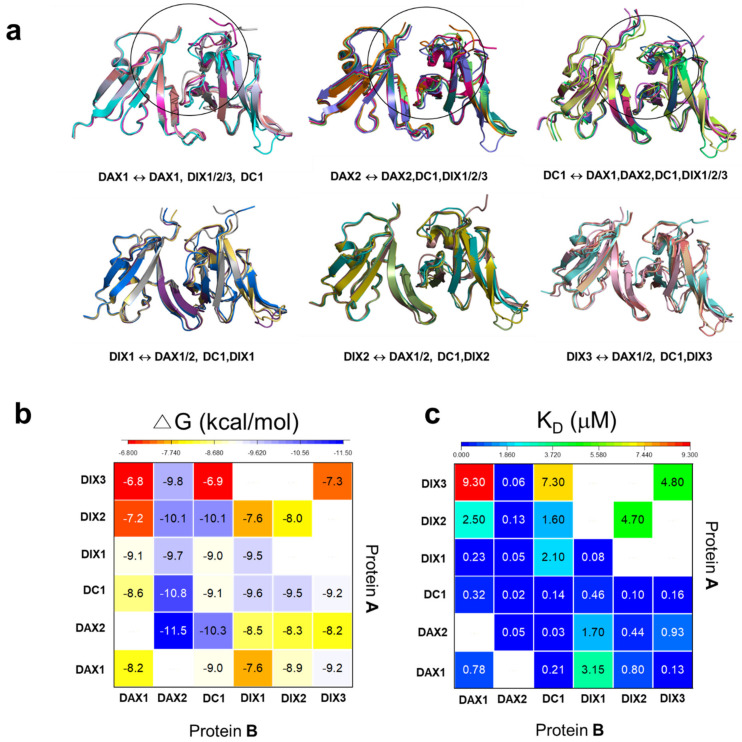
The 28 complex structures of the DIX domains predicted by AlphaFold2: (**a**) The superposition of the complex structures shows the head-to-tail interface contact. DAX1/2, DIX1/2/3, and DC1 represent the DIX domain of the Axin1/2, Dvl 1/2/3, and Ccd1 Dix domain, respectively. For example, DAX1-DAX1 defines the tail region of Axin1 DIX (protein A) as interacting with the head region of the Axin1 DIX domain (protein B). (**b**) The binding energies (ΔG, kcal/mol) and (**c**) binding affinities (K_D_, μM) of the homodimers and heterodimers of the DIX domains. The selected highest pLDDT value complex structure that AlphaFold2 predicted was optimized by the amber force field implemented in the HyperChem 8.0 software. The optimized 3D structures of DIX domains were used to calculate the binding affinity of the complex with the PRODIGY web server [48].

To understand the binding mode of DIX-mediated complexes, we generated all 6 possible homodimers and 22 heterodimers of the DIX domain using two methods introduced in the computational method section (SI Figure S1). For example, we obtained the human Axin1 homodimers using the residue index method (Method 1, Axin1 DIX: Axin1 DIX) and the 6x GGS linker method (Method 2, Axin1 DIX-(GGS)_6_-Axin1 DIX). Generally, both methods provided the same results, showing the head-to-tail interaction of homodimers. We found, however, that the predicted homodimers DAX2 (Axin2 DIX) and DC1 (Ccd1 DIX) showed head-to-head interface contact with high PAE values (low confidence) if we used the first method (SI Figure S3a). We found that the second method has another benefit. For example, the sequences of DAX1-(GGS)_6_-DIX2 and DIX2-(GGS)_6_-DAX1 produced two different complexes: the DAX1-(GGS)_6_-DIX2 complex shows the interaction between the tail interface of DAX1 and the head interface of DIX2. The DIX2-(GGS)_6_-DAX1 complex structure shows the interaction between the tail interface of DIX2, which makes contact with the head interface of DAX1. All predicted homodimers and heterodimers have similar structural features, i.e., head-to-tail interaction. However, the binding regions (especially head interfaces) of the complexes are relatively flexible (Figure 2a). In addition, the complex structures of homodimers and heterodimers support the previous conclusion that DIX-mediated homotypic and heterotypic interactions share the same head and tail residues [11].

For comparison, we used SWISS-MODEL [55] and GalaxyWEB [51], which used template-based homology modeling or ab initio Docking to predict the homodimers and heterodimers of the DIX domain. However, we observed that both methods failed to predict the homodimers and heterodimers (SI Figure S7). These results may also support the superior performance of AF2 in predicting the homodimers and heterodimers of DIX-mediated interactions. When we used AF2-predicted structures, the HawkDock [52] could correctly generate the complex structures of the DIX domains.

### 3.2. Evaluation of the Binding Affinity of DIX Domains

Because of the high accuracy of the AF2-predicted complex, we wondered whether we could calculate the binding energy of the complexes using the PRODIGY [48]. We thus evaluated the accuracy of the PRODIGY method developed to calculate the free binding energies (ΔG, kcal/mol) and binding affinities (K_D_) (Figure 2b,c; SI Figure S8 and Table S3). The results showed we could obtain better K_D_ values once we optimized the selected top-ranked complex before calculating the binding affinity. For comparison, the HAWKDOCK was used to obtain the binding energies of the DIX domain using docking and molecular mechanics/the generalized MM/GBSA method [52] (SI Table S3)

A handful of binding affinities of DIX domains are available, although there were some discrepancies between the experiments. The reported K_D_ value of the DAX mutant interactions between DAX1-M3 and DAX1-M2 was 45 μM using fluorescence (See Table 1 notes). However, using SEC-MALS analysis, Kan et al. [38] estimated the K_D_ value of the wild-type DAX1 dimers and oligomers, showing that the intrinsic K_D_ value of the wild-type DAX1 homodimer was 0.24 μM, and the apparent K_D_ values of the wild-type DAX1 oligomers were 0.9 μM. Since the binding affinities of the homotypic DIX-meditated interaction were used to explain the mechanism of action in the proposed models of Wnt signaling [12,38,41], we generated both complexes using AF2 and then calculated the binding affinity of DAX1↔DAX1 and DAX1-M3(I758A/R761D) ↔ DAX1-M2(V800A/F801A). Remarkably, the predicted K_D_ value of the wild-type DAX1 homodimer using PRODIGY is 0.78 ± 0.30 μM, consistent with the SEC-MALS analysis. The calculated K_D_ value of the DAX1-M3 ↔ DAX1-M2 interaction is 1.2 μM, which is close to the wild-type homotypic DAX interaction. We also calculated the K_D_ value of the wild-type and mutant DIX homodimers (SI Table S3). Previously, using analytical ultracentrifugation, the binding affinity of DIX2 self-association was estimated to be 5–10 μM [11]. Consistent with this result, Schwarz-Romond et al. [16] reported that the K_D_ value of DIX2-M4(Y27D) and DIX2-M2(V67A/K68A) was estimated to be 4.9 μM using NMR spectroscopy. Using the PRODIGY method, the predicted K_D_ value of DIX2-M3 and DIX2-M4 homodimer is 4.7 ± 4.7 μM, and the predicted K_D_ value of the wild-type DIX self-association is 4.8 μM (Table 1). Since the K_D_ values calculated by the PRODIGY server are rather close to the estimated K_D_ value determined by experiments, we further calculated the binding affinities of the DIX homodimers and heterodimers using the PRODIGY (Table 1 and SI Table S3).

### 3.3. Prediction of Binding Affinities of Homodimers

The predicted homodimers of the DIX domains show high affinities in the range of 0.021 to 78 μM (Table 1 and Figure 2b,c). The binding affinity of DAX2-DAX2 self-association (K_D_ = 0.05 ± 0.07 μM) is calculated to be 16× more potent than that of the DAX1 homodimer (K_D_ = 0.78 ± 0.30 μM). For Dishevelled, interestingly, the DIX1-DIX1 interaction is much stronger than the DIX2-DIX2 (or DIX3-DIX3) homotypic interaction. The binding affinity of Ccd1 DIX(DC1) self-association is comparable to the DAX1 homodimer. Thus, the relative order of homodimers’ binding strengths was DAX2 > DC1 > DIX1 >> DIX2~DIX3. The weak interaction of DIX2 and DIX3 might explain the dynamic of Dvl oligomerization [11,16]. The different binding affinities of the Axin1 and Axin2 DIX domains and Dvl1 DIX and Dvl2/3 DIX domains partly imply that these protein paralogs may have a distinct role in Wnt signaling [24,25], although the DIX-mediated interactions might not explain the mechanism fully. However, the strong DAX-DAX homotypic interaction may explain why cytoplasmic Dvl may prevent the disruption of DAX-DAX interactions (Figure 2b) [38].

### 3.4. Prediction of Binding Affinities of Heterodimers

Structural and biochemical studies revealed the direct interaction of the DIX domains of three proteins, Axin, Dvl, and Ccd1 [11,16,18,19,20,35,36,38,56], showing that these interactions are critical to regulating canonical Wnt signaling. In general, the binding affinities of heterodimers are predicted to be stronger than those of the DIX2-DIX2 and DIX3-DIX3 homodimers but weaker than those of the homodimers of DAX1-DAX1 and DAX2-DAX2.

For the heterodimers, we considered two binding modes: the interaction between the tail interface of Protein A and the head interface of Protein B (Protein A–Protein B) and vice versa (Protein B–Protein A). For example, DAX-DIX represents that the tail interface of DAX interacting with the head interface of DIX.

For the DAX1-DIX1/2/3 interaction, the tail interface of Axin1 DIX (DAX1) contacts with the head interface of DIX3 (ΔG = −9.2 kcal/mol) more favorably than that of DIX2 (ΔG = −8.9 kcal/mol) and DIX1 (ΔG= −7.6 kcal/mol). For the DIX1/2/3-DAX1 complex, the binding of DIX1 (ΔG = –9.1 kcal/mol) is more favorable than that of DIX2 (ΔG= –7.2 kcal/mol) and DIX3 (ΔG= –6.8 kcal/mol) with DAX1. Remarkably, the binding affinity (K_D_) of the heterolytic DIX2/3-DAX1 interaction is 10× ~ 70× weaker than the DAX1-DIX2/3 interaction (Figure 2c and Table 1). The predicted results support a study that reported that DAX1 might control the oligomerization of the DIX2 [38]. Kan et al. [38] found that DAX1 binds to the ends of Dvl oligomers, indicating that there are likely to be roughly matched numbers of Axin and Dvl associated with the activated receptors. The overall results imply that the difference in binding affinity between DAX-DIX and DIX-DAX controls the size of Dvl oligomers.

The binding affinities (K_D_) of heterotypic DAX1(or DAX2)-DIX1(DIX2 or DIX/3) or DIX1(DIX2 or DIX3)-DAX1(or DAX2) interactions are found in the range of 0.05~9.30 μM, depending on the Dvl paralogs. Thus, the mechanism of action of the Axin-Dvl interaction is complicated. The homolytic DAX1-DAX1 (K_D_ = 0.78 μM) interaction is weaker than the heterotypic DIX1-DAX1 (K_D_ = 0.23 μM) and DAX1-DIX3 (0.13 μM) interactions but stronger than DAX1-DIX1 (K_D_ = 3.15 μM), DIX2-DAX1(K_D_ = 2.50 μM), and DIX3-DAX1 (K_D_ = 9.30 μM). The weak homolytic DIX2-DIX2 (K_D_ = 4.70 μM) or DIX3-DIX3 (K_D_ = 4.80 μM) interaction may not disrupt the homolytic DAX1-DAX1 (K_D_ = 0.78 μM) and DAX2-DAX2 (K_D_ = 0.05 μM). This implies that other factors, such as liquid–liquid phase separation (LLPS) and the concentration of DIX-containing proteins *in vivo*, may affect the binding mode of Axin and Dvl proteins [13,38,57].

Compared to the DAX1-DIX1(DIX2 or DIX3), the DAX2-DIX1(DIX2 or DIX3) complex shows different binding preferences. The DAX2-DIX2 or DAX2-DIX3 interaction is less potent than the heterolytic DIX2-DAX2 or DIX3-DAX2 heterotypic interaction, similar to the homolytic DAX2-DAX2 dimer. Notably, DAX2-DIX1 binding (K_D_ = 1.70 μM) is about 34× weaker than the DIX1-DAX2 interaction (K_D_ = 0.05 μM). These results also imply that Axin and Dvl paralogs, at least, may have a distinct role in Wnt signaling in terms of DIX1-mediated interactions.

Ccd1 DIX (DC1) may interact with the DAX1(or DAX2) and DIX1(DIX2 or DIX3) domains in vivo and in vitro [19,20]. The predicted binding affinity of DAX1-DC1 (K_D_~0.21 μM) is lower than that of DC1-DAX1 (K_D_ = 0.32 μM) (Figure 2c). The result indicates that the tail interface of DAX1 strongly interacts with the head interface of DC1. For the DAX2 protein, however, the predicted binding affinity of DAX2-DC1 (K_D_~0.03 μM) is similar to that of DC1-DAX2 (K_D_~0.02 μM). Compared to the DAX1-DC1 interaction, the DAX2-DC1 interaction is more potent, implying that a distinct mechanism of DAX2 or DAX1 may exist.

Compared to the DC1-DAX1 (DC1-DAX2) or DAX1-DC1(DAX2-DC1) interaction, the binding affinity of DIX1(DIX2 or DIX3)-DC1 is 10× weaker than the DC1-DIX1(DIX2 or DIX3) interaction. The predicted binding affinities of DC1-DIX1 (DIX2 or DIX3) are in the range of 0.10~0.46 μM (Figure 2c, SI Table S3), suggesting that the tail interface of DC1 prefers the head interface of DIX1 (DIX2 or DIX3). Consistent with this discovery, NMR- and GST-mediated pulldown assays showed a heteromeric interaction between DC1-DIX2 but not DIX2-DC1 [19]. Since the binding affinity of the heterolytic DC1-DIX1(DIX2 or DIX3) interaction is stronger than the DIX1(DIX2, or DIX3) homodimer interaction, we expect that DC1 may also disrupt DIX oligomerization, which is similar to the role of DAX1 as Wnt signaling is activated [38].

The binding energies of DIX domains were also obtained using the HawkDock server [52]. For comparison, we obtained the binding energies of DIX domains using an MM/GBSA calculation (SI Table S3). The binding energies of DAX1-DAX1, DIX2-DIX2, DAX1-DIX2, and DIX2-DAX1 are –116.13, –99.38, –109.38, and –80.61 kcal/mol, respectively. Although the predicted ΔG value of the DIX domain interactions is very high compared to the reported experimental values, the relative binding ability of the DIX domains is the same as the PRODIGY’s prediction, such as DAX1-DAX1 > DAX1-DIX2 > DIX2-DIX2 > DIX2-DAX1 (SI Table S3).

### 3.5. Analysis of the Interface Residues Involved in DIX-DIX Interactions

We analyzed the interface residues involved in DIX-DIX interactions using the PRODIGY server to understand the different binding affinities (SI Figure S9). The residues in the binding interfaces, hydrogen bonds, and salt bridges of the DIX-mediated interaction predicted by AF2 were analyzed using the PDBePISA (Proteins, Interfaces, Structures and Assemblies) website (SI Tables S5 and S6) [58,59,60]. The detailed information is deposited in Figshare, https://doi.org/10.6084/m9.figshare.27042208, accessed on 11 October 2022).

The residues involved in the interaction are in the head (strands β1 and β2 and C terminus of α-helix) and tail interface (strands β3 and β4) of the DIX domains, suggesting that the residues are engaged in both homodimers and heterodimers with relatively few differences (Figure 1a). Notably, the various residues involved in binding are found at the end of the β5 regions and the C terminus of α-helix (SI Tables S6 and S7). PDBePISA analysis indicates that no hydrogen bond and salt bridges are found in this interface for the DIX2-DAX1, DIX2-DC1, DIX3-DIX3, DIX3-DAX1, and DIX3-DC1 due to the nature of hydrophobic interactions (SI Figure S10). Although it is likely not simple to rationalize the different binding affinity trends in the DIX protein’s paralogs based on their sequence differences, our results would provide essential information on the DIX-mediated interactions in Wnt signaling [61,62]. Since AF2-predicted complex structures with the interface’s information of DIX-mediated interactions were analyzed, the systematic mutagenesis studies identified critical residues for the intermolecular interactions that would help elucidate the functions of DIX-containing proteins.

### 3.6. Molecular Mechanism by Which DIX-Mediated Wnt Signaling Occurs

When the Wnt signal is on, the Wnt protein binds to the FZD and LRP5/6 proteins. At the plasma membrane, the Wnt-FZD-LRP5/6 complex [9,14] may recruit (i) the condensate Dvl protein [13] or (ii) the condensate Axin protein [63,64] by an unclear mechanism. The first Wnt activation model is that the recruited condensated Dvl proteins form the signalosome containing Wnt-Fzd-LRP5/6-Dvl [38,65] and then consequently interact with Axin through the DIX-mediated interaction. GSK-3β and CK1α in the destruction complex phosphorylate the C-terminal of LRP5/6 [66,67], which is critical to the binding to the Axin protein [33]. The second Wnt activation model is that the Wnt-FZD-LRP5/6 complex may recruit the condensate Axin protein to the plasma membrane [63] and then bring the destruction complex to the membrane. LRP5/6 C-terminus protein is phosphorylated by GSK-3β and CK1α [66,67]. The LRP5/6-binding Axin protein may bring the condensate Dvl protein to the plasma membrane through the DIX-mediated interaction. The two molecular mechanisms are similar, but the reaction order at the plasma membrane level in Wnt signaling is under debate [68,69]. Because recent studies showed that the intrinsically disordered regions (IDRs) in Axin and Dvl proteins are important in regulating the Wnt signaling pathways [70], the role of IDRs in the DIX-mediated interactions is also interesting. In addition, the roles of other conserved domains found in Dvl and Axin in DIX-mediated interactions in the Wnt signaling pathway [35] remain to be explored.

## 4. Conclusions

Characterizing the protein–protein interactions and specificity is crucial to understanding the mechanism of action in Wnt signaling. Because of the diversity of proteins in cells and challenges in the lab, theoretical approaches such as homology models, docking, and molecular dynamics have been employed [71,72]. Herein, we used AF2 to generate the homodimers and heterodimers of DIX domains without any templates, showing the superior performance of AF2. The 32 complex structures of the wild-type DIX domains, including 4 DIX mutants, showed similar structural features through head-to-tail interactions. The binding affinities of the DIX-mediated interactions, determined using the PRODIGY, were obtained based on the AF2-predicted and optimized structures. The predicted binding affinities (K_D_) of the DIX-DIX interaction are, at best, comparable to the reported experimental data. The predicted K_D_ values of the DIX homodimers and heterodimers partially explain the molecular mechanism underlying how Axin1/2, Dvl1/2/3, and Ccd1 regulate intracellular Wnt signaling transduction. We identified the residues found in the head and tail interfaces of the AF2-predicted DIX complexes, which may guide further biological experiments. Our results support the idea that AF2 can explore uncovered protein–protein interactions at the atomic level within a reasonable time frame and, most importantly, with high accuracy. Since the AF2-powered ColabFold is easy to use and fast to predict the dimers, we believe that AF2 can explore the unveiled biological phenomena in Wnt signaling [73] and will guide biological experiments based on structural information. We suggest that the methods used here can be employed for education and research (Supplementary Figure S1).

## Figures and Tables

**Table 1:** Thermodynamic parameters (ΔG, in kcal/mol; K_D_ in μM) of homodimers and heterodimers of DIX domains ^a^.

Protein A	Protein B	^b^ ΔG	^c^ K_D_	Protein A	Protein B	^a^ ΔG	^b^ K_D_
DAX1	DAX1 ^d^	−8.4 ± 0.2	0.78 ± 0.30	DIX1	DIX1	−9.8 ± 0.4	0.084 ± 0.05
DAX1-M3	DAX1-M2 ^d^	−8.1	1.2	DIX2	DIX2 ^d^	−7.5 ± 0.8	4.7 ± 4.7
DAX2	DAX2	−10.6 ± 1.3	0.05 ± 0.07	DIX2-M4	DIX2-M2 ^d^	−7.3	4.7
DC1	DC1	−9.4 ± 0.4	0.14 ± 0.08	DIX3	DIX3	−7.3	4.8
DAX1	DIX1	−7.5 ± 0.1	3.15 ± 0.64	DIX1	DAX1	−9.1 ± 0.1	0.23 ± 0.01
DAX1	DIX2	−8.5 ± 0.6	0.8 ± 0.7	DIX2	DAX1	−8.2 ± 1.3	2.5 ± 3.3
DAX1-M3	DIX2-M2 ^d^	−8.1 ± 0.7	1.5 ± 1.4	DIX2-M4	DAX1-M2 ^d^	−6.6 ± 0.3	14.4 ± 6.5
DAX1	DIX3	−9.5 ± 0.4	0.13 ± 0.08	DIX3	DAX1	−6.9 ± 0.1	9.3 ± 0.4
DAX2	DIX1	−8.1 ± 0.6	1.7 ± 1.7	DIX1	DAX2	−10.2 ± 0.6	0.05 ± 0.05
DAX2	DIX2	−9.0 ± 1.0	0.44 ± 0.52	DIX2	DAX2	−9.6 ± 0.7	0.13 ± 0.12
DAX2	DIX3	−8.2	0.930	DIX3	DAX2	−9.8 ± 1.1	0.060
DAX1	DC1	−9.2 ± 0.3	0.21 ± 0.09	DC1	DAX1	−9.0 ± 0.5	0.32 ± 0.25
DAX2	DC1	−10.2 ± 0.1	0.03 ± 0.01	DC1	DAX2	−10.7 ± 0.2	0.02 ± 0.01
DIX1	DC1	−8.2 ± 0.8	2.1 ± 2.6	DC1	DIX1	−9.0 ± 0.9	0.46 ± 0.53
DIX2	DC1	−8.2 ± 0.1	1.6 ± 1.7	DC1	DIX2	−9.5	0.100
DIX3	DC1	−7.0 ± 0.1	7.3 ± 1.0	DC1	DIX3	−9.3 ± 0.1	0.16 ± 0.02

^a^ Thermodynamics parameters were calculated by using the PRODIGY server. ^b^ Average ΔG value calculated from the AlphaFold2-predicted complex by two methods (see also SI Table S3). ^c^ Average K_D_ values were calculated using ΔG = −RT ln K_D_, T = 298K [48]. ^d^ Experimentally estimated K_D_ values: DAX1↔ DAX1, K_D_ = 0.24 μM [38]; DAX1-M3↔DAX1-M2, K_D_ = 45 μM [11]; DIX2↔DIX2, K_D_ = 5–20 μM [11], DIX2-M4↔DIX-M2, K_D_ = 4.9 μM [38]; DAX1-M3↔DIX2-M2, K_D_ = 24 μM [38]; DIX2-M4↔DAX1-M2, K_D_ = 9 μM [38].

## Data Availability

The data presented in this study are available in the article or Supplementary Material (Figshare, https://doi.org/10.6084/m9.figshare.27042208).

## Supplementary Materials

The following supporting information can be downloaded at: https://www.mdpi.com/article/10.3390/cells13191646/s1, The supporting information contains Figures S1–S10 and Tables S1–S6. The AF2-predicted structures of the DIX-DIX complex and the detailed binding interfaces, hydrogen bonds, and salt bridges of the DIX-mediated interactions analyzed by using PDBePISA were deposited in Figshare, https://doi.org/10.6084/m9.figshare.27042208. Further inquiries can be directed to the corresponding authors.

## References

[1] Zhao Y., Yang Z.Q., Wang Y., Miao Y., Liu Y., Dai S.D., Han Y., Wang E.H. (2010). Dishevelled-1 and dishevelled-3 affect cell invasion mainly through canonical and noncanonical Wnt pathway, respectively, and associate with poor prognosis in nonsmall cell lung cancer. Mol. Carcinog..

[2] Zhou G., Ye J., Sun L., Zhang Z., Feng J. (2016). Overexpression of Dishevelled-2 contributes to proliferation and migration of human esophageal squamous cell carcinoma. J. Mol. Histol..

[3] Liao W.Y., Hsu C.C., Chan T.S., Yen C.J., Chen W.Y., Pan H.W., Tsai K.K. (2020). Dishevelled 1-Regulated Superpotent Cancer Stem Cells Mediate Wnt Heterogeneity and Tumor Progression in Hepatocellular Carcinoma. Stem Cell Rep..

[4] Khan A.S., Hojjat-Farsangi M., Daneshmanesh A.H., Hansson L., Kokhaei P., Osterborg A., Mellstedt H., Moshfegh A. (2016). Dishevelled proteins are significantly upregulated in chronic lymphocytic leukaemia. Tumour Biol..

[5] Kim P.J., Park J.Y., Kim H.G., Cho Y.M., Go H. (2017). Dishevelled segment polarity protein 3 (DVL3): A novel and easily applicable recurrence predictor in localised prostate adenocarcinoma. BJU Int..

[6] Mei J., Yang X., Xia D., Zhou W., Gu D., Wang H., Liu C. (2020). Systematic summarization of the expression profiles and prognostic roles of the dishevelled gene family in hepatocellular carcinoma. Mol. Genet. Genom. Med..

[7] Du W., Menjivar R.E., Donahue K.L., Kadiyala P., Velez-Delgado A., Brown K.L., Watkoske H.R., He X., Carpenter E.S., Angeles C.V. (2023). WNT signaling in the tumor microenvironment promotes immunosuppression in murine pancreatic cancer. J. Exp. Med..

[8] Micka M., Bryja V. (2021). Can We Pharmacologically Target Dishevelled: The Key Signal Transducer in the Wnt Pathways?. Handb. Exp. Pharmacol..

[9] Bilic J., Huang Y.L., Davidson G., Zimmermann T., Cruciat C.M., Bienz M., Niehrs C. (2007). Wnt induces LRP6 signalosomes and promotes dishevelled-dependent LRP6 phosphorylation. Science.

[10] Metcalfe C., Mendoza-Topaz C., Mieszczanek J., Bienz M. (2010). Stability elements in the LRP6 cytoplasmic tail confer efficient signalling upon DIX-dependent polymerization. J. Cell Sci..

[11] Fiedler M., Mendoza-Topaz C., Rutherford T.J., Mieszczanek J., Bienz M. (2011). Dishevelled interacts with the DIX domain polymerization interface of Axin to interfere with its function in down-regulating β-catenin. Proc. Natl. Acad. Sci. USA.

[12] Yamanishi K., Fiedler M., Terawaki S.I., Higuchi Y., Bienz M., Shibata N. (2019). A direct heterotypic interaction between the DIX domains of Dishevelled and Axin mediates signaling to beta-catenin. Sci. Signal.

[13] Kang K., Shi Q., Wang X., Chen Y.-G. (2022). Dishevelled phase separation promotes Wnt signalosome assembly and destruction complex disassembly. J. Cell Biol..

[14] Tsutsumi N., Hwang S., Waghray D., Hansen S., Jude K.M., Wang N., Miao Y., Glassman C.R., Caveney N.A., Janda C.Y. (2023). Structure of the Wnt–Frizzled–LRP6 initiation complex reveals the basis for coreceptor discrimination. Proc. Natl. Acad. Sci. USA.

[15] Sharma M., Castro-Piedras I., Simmons G.E., Pruitt K. (2018). Dishevelled: A masterful conductor of complex Wnt signals. Cell. Signal..

[16] Schwarz-Romond T., Fiedler M., Shibata N., Butler P.J., Kikuchi A., Higuchi Y., Bienz M. (2007). The DIX domain of Dishevelled confers Wnt signaling by dynamic polymerization. Nat. Struct. Mol. Biol..

[17] Madrzak J., Fiedler M., Johnson C.M., Ewan R., Knebel A., Bienz M., Chin J.W. (2015). Ubiquitination of the Dishevelled DIX domain blocks its head-to-tail polymerization. Nat. Commun..

[18] Shiomi K., Uchida H., Keino-Masu K., Masu M. (2003). Ccd1, a novel protein with a DIX domain, is a positive regulator in the Wnt signaling during zebrafish neural patterning. Curr. Biol..

[19] Liu Y.T., Dan Q.J., Wang J., Feng Y., Chen L., Liang J., Li Q., Lin S.C., Wang Z.X., Wu J.W. (2011). Molecular basis of Wnt activation via the DIX domain protein Ccd1. J. Biol. Chem..

[20] Terawaki S.I., Fujita S., Katsutani T., Shiomi K., Keino-Masu K., Masu M., Wakamatsu K., Shibata N., Higuchi Y. (2017). Structural basis for Ccd1 auto-inhibition in the Wnt pathway through homomerization of the DIX domain. Sci. Rep..

[21] Itoh K., Antipova A., Ratcliffe M.J., Sokol S. (2000). Interaction of dishevelled and Xenopus axin-related protein is required for wnt signal transduction. Mol. Cell Biol..

[22] Kishida S., Yamamoto H., Hino S., Ikeda S., Kishida M., Kikuchi A. (1999). DIX domains of Dvl and axin are necessary for protein interactions and their ability to regulate beta-catenin stability. Mol. Cell Biol..

[23] Wong C.K., Luo W., Deng Y., Zou H., Ye Z., Lin S.-C. (2004). The DIX Domain Protein Coiled-coil-DIX1 Inhibits c-Jun N-terminal Kinase Activation by Axin and Dishevelled through Distinct Mechanisms. J. Biol. Chem..

[24] Gentzel M., Schambony A. (2017). Dishevelled Paralogs in Vertebrate Development: Redundant or Distinct?. Front. Cell Dev. Biol..

[25] Paclikova P., Radaszkiewicz T.W., Potesil D., Harnos J., Zdrahal Z., Bryja V. (2021). Roles of individual human Dishevelled paralogs in the Wnt signalling pathways. Cell. Signal..

[26] Arnold H.K., Zhang X., Daniel C.J., Tibbitts D., Escamilla-Powers J., Farrell A., Tokarz S., Morgan C., Sears R.C. (2009). The Axin1 scaffold protein promotes formation of a degradation complex for c-Myc. EMBO J..

[27] Chia I.V., Costantini F. (2005). Mouse axin and axin2/conductin proteins are functionally equivalent in vivo. Mol. Cell Biol..

[28] Jho E.H., Zhang T., Domon C., Joo C.K., Freund J.N., Costantini F. (2002). Wnt/beta-catenin/Tcf signaling induces the transcription of Axin2, a negative regulator of the signaling pathway. Mol. Cell Biol..

[29] Gai Z., Wang Y., Tian L., Gong G., Zhao J. (2021). Whole Genome Level Analysis of the Wnt and DIX Gene Families in Mice and Their Coordination Relationship in Regulating Cardiac Hypertrophy. Front. Genet..

[30] Boligala G.P., Yang M.V., van Wunnik J.C., Pruitt K. (2022). Nuclear Dishevelled: An enigmatic role in governing cell fate and Wnt signaling. Biochim. Biophys. Acta BBA Mol. Cell Res..

[31] Lee H.J., Shi D.L., Zheng J.J. (2015). Conformational change of Dishevelled plays a key regulatory role in the Wnt signaling pathways. eLife.

[32] Qi J., Lee H.-J., Saquet A., Cheng X.-N., Shao M., Zheng J.J., Shi D.-L. (2017). Autoinhibition of Dishevelled protein regulated by its extreme C terminus plays a distinct role in Wnt/β-catenin and Wnt/planar cell polarity (PCP) signaling pathways. J. Biol. Chem..

[33] Harnoš J., Cañizal M.C.A., Jurásek M., Kumar J., Holler C., Schambony A., Hanáková K., Bernatík O., Zdráhal Z., Gömöryová K. (2019). Dishevelled-3 conformation dynamics analyzed by FRET-based biosensors reveals a key role of casein kinase 1. Nat. Commun..

[34] Hanáková K., Bernatík O., Kravec M., Micka M., Kumar J., Harnoš J., Ovesná P., Paclíková P., Rádsetoulal M., Potěšil D. (2019). Comparative phosphorylation map of Dishevelled 3 links phospho-signatures to biological outputs. Cell Commun. Signal..

[35] Schubert A., Voloshanenko O., Ragaller F., Gmach P., Kranz D., Scheeder C., Miersch T., Schulz M., Trumper L., Binder C. (2022). Superresolution microscopy localizes endogenous Dvl2 to Wnt signaling-responsive biomolecular condensates. Proc. Natl. Acad. Sci. USA.

[36] Terawaki S., Yano K., Katsutani T., Shiomi K., Keino-Masu K., Masu M., Shomura Y., Komori H., Shibata N., Higuchi Y. (2011). Crystallographic characterization of the DIX domain of the Wnt signalling positive regulator Ccd1. Acta Crystallogr. Sect. F Struct. Biol. Cryst. Commun..

[37] Yamanishi K., Kumano W., Terawaki S.I., Higuchi Y., Shibata N. (2019). Head-to-Tail Complex of Dishevelled and Axin-DIX Domains: Expression, Purification, Crystallographic Studies and Packing Analysis. Protein Pept. Lett..

[38] Kan W., Enos M.D., Korkmazhan E., Muennich S., Chen D.H., Gammons M.V., Vasishtha M., Bienz M., Dunn A.R., Skiniotis G. (2020). Limited dishevelled/Axin oligomerization determines efficiency of Wnt/β-catenin signal transduction. eLife.

[39] Shibata N., Tomimoto Y., Hanamura T., Yamamoto R., Ueda M., Ueda Y., Mizuno N., Ogata H., Komori H., Shomura Y. (2007). Crystallization and preliminary X-ray crystallographic studies of the axin DIX domain. Acta Crystallogr. Sect. F Struct. Biol. Cryst. Commun..

[40] Yamanishi K., Sin Y., Terawaki S.I., Higuchi Y., Shibata N. (2019). High-resolution structure of a Y27W mutant of the Dishevelled2 DIX domain. Acta Crystallogr. F Struct. Biol. Commun..

[41] Ehebauer M.T., Arias A.M. (2009). The structural and functional determinants of the Axin and Dishevelled DIX domains. BMC Struct. Biol..

[42] Jumper J., Evans R., Pritzel A., Green T., Figurnov M., Ronneberger O., Tunyasuvunakool K., Bates R., Zidek A., Potapenko A. (2021). Highly accurate protein structure prediction with AlphaFold. Nature.

[43] Varadi M., Anyango S., Deshpande M., Nair S., Natassia C., Yordanova G., Yuan D., Stroe O., Wood G., Laydon A. (2022). AlphaFold Protein Structure Database: Massively expanding the structural coverage of protein-sequence space with high-accuracy models. Nucleic Acids Res..

[44] Wang L., Wen Z., Liu S.-W., Zhang L., Finley C., Lee H.-J., Fan H.-J.S. (2024). Overview of AlphaFold2 and breakthroughs in overcoming its limitations. Comput. Biol. Med..

[45] Bryant P., Pozzati G., Elofsson A. (2022). Improved prediction of protein-protein interactions using AlphaFold2. Nat. Commun..

[46] Mirdita M., Schütze K., Moriwaki Y., Heo L., Ovchinnikov S., Steinegger M. (2022). ColabFold: Making protein folding accessible to all. Nat. Methods.

[47] Skolnick J., Gao M., Zhou H., Singh S. (2021). AlphaFold 2: Why It Works and Its Implications for Understanding the Relationships of Protein Sequence, Structure, and Function. J. Chem. Inf. Model..

[48] Xue L.C., Rodrigues J.P., Kastritis P.L., Bonvin A.M., Vangone A. (2016). PRODIGY: A web server for predicting the binding affinity of protein-protein complexes. Bioinformatics.

[49] Vangone A., Bonvin A.M. (2015). Contacts-based prediction of binding affinity in protein-protein complexes. eLife.

[50] Froimowitz M. (1993). HyperChem: A software package for computational chemistry and molecular modeling. Biotechniques.

[51] Ko J., Park H., Heo L., Seok C. (2012). GalaxyWEB server for protein structure prediction and refinement. Nucleic Acids Res..

[52] Weng G., Wang E., Wang Z., Liu H., Zhu F., Li D., Hou T. (2019). HawkDock: A web server to predict and analyze the protein-protein complex based on computational docking and MM/GBSA. Nucleic Acids Res..

[53] Saldano T., Escobedo N., Marchetti J., Zea D.J., Mac Donagh J., Velez Rueda A.J., Gonik E., Garcia Melani A., Novomisky Nechcoff J., Salas M.N. (2022). Impact of protein conformational diversity on AlphaFold predictions. Bioinformatics.

[54] Tunyasuvunakool K., Adler J., Wu Z., Green T., Zielinski M., Zidek A., Bridgland A., Cowie A., Meyer C., Laydon A. (2021). Highly accurate protein structure prediction for the human proteome. Nature.

[55] Shankaracharya A.M., Vidyarthi A.S. (2012). SWIFT MODELLER v2.0: A platform-independent GUI for homology modeling. J. Mol. Model..

[56] Ma W., Chen M., Kang H., Steinhart Z., Angers S., He X., Kirschner M.W. (2020). Single-molecule dynamics of Dishevelled at the plasma membrane and Wnt pathway activation. Proc. Natl. Acad. Sci. USA.

[57] Babcock R.L., Pruitt K. (2022). Letting go: Dishevelled phase separation recruits Axin to stabilize β-catenin. J. Cell Biol..

[58] Krissinel E., Henrick K. (2007). Inference of macromolecular assemblies from crystalline state. J. Mol. Biol..

[59] Krissinel E., Hendrick K., Berthold M.R., Glen R.C., Diederichs K., Kohlbacher O., Fischer I. (2005). Detection of Protein Assemblies in Crystals. Computational Life Sciences.

[60] Krissinel E. (2010). Crystal contacts as nature’s docking solutions. J. Comput. Chem..

[61] Genheden S., Ryde U. (2015). The MM/PBSA and MM/GBSA methods to estimate ligand-binding affinities. Expert. Opin. Drug Discov..

[62] Wang C., Greene D., Xiao L., Qi R., Luo R. (2017). Recent Developments and Applications of the MMPBSA Method. Front. Mol. Biosci..

[63] Nong J., Kang K., Shi Q., Zhu X., Tao Q., Chen Y.G. (2021). Phase separation of Axin organizes the β-catenin destruction complex. J. Cell Biol..

[64] Schaefer K.N., Peifer M. (2019). Wnt/Beta-Catenin Signaling Regulation and a Role for Biomolecular Condensates. Dev. Cell.

[65] Beitia G.J., Rutherford T.J., Freund S.M.V., Pelham H.R., Bienz M., Gammons M.V. (2021). Regulation of Dishevelled DEP domain swapping by conserved phosphorylation sites. Proc. Natl. Acad. Sci. USA.

[66] Zeng X., Huang H., Tamai K., Zhang X., Harada Y., Yokota C., Almeida K., Wang J., Doble B., Woodgett J. (2008). Initiation of Wnt signaling: Control of Wnt coreceptor Lrp6 phosphorylation/activation via frizzled, dishevelled and axin functions. Development.

[67] Zeng X., Tamai K., Doble B., Li S., Huang H., Habas R., Okamura H., Woodgett J., He X. (2005). A dual-kinase mechanism for Wnt co-receptor phosphorylation and activation. Nature.

[68] Tacchelly-Benites O., Wang Z., Yang E., Lee E., Ahmed Y. (2013). Toggling a conformational switch in Wnt/β-catenin signaling: Regulation of Axin phosphorylation. The phosphorylation state of Axin controls its scaffold function in two Wnt pathway protein complexes. Bioessays.

[69] Mannava A.G., Tolwinski N.S. (2015). Membrane bound GSK-3 activates Wnt signaling through disheveled and arrow. PLoS ONE.

[70] Vamadevan V., Chaudhary N., Maddika S. (2022). Ubiquitin-assisted phase separation of dishevelled-2 promotes Wnt signalling. J. Cell Sci..

[71] Agostino M., Pohl S.O., Dharmarajan A. (2017). Structure-based prediction of Wnt binding affinities for Frizzled-type cysteine-rich domains. J. Biol. Chem..

[72] Agostino M., Pohl S.O. (2019). Wnt Binding Affinity Prediction for Putative Frizzled-Type Cysteine-Rich Domains. Int. J. Mol. Sci..

[73] Agostino M., Pohl S.O. (2020). The structural biology of canonical Wnt signalling. Biochem. Soc. Trans..

