# Supplementary material for: Exploring DIX-DIX Homo- and Hetero-Oligomers in Wnt Signaling with AlphaFold2
Source: Cells. 2024 Oct 3;13(19):1646. doi: 10.3390/cells13191646 (PMC11475284; doi:10.3390/cells13191646)
Supplement: Supplementary file 1 [file cells-13-01646-s001.zip › cells-3181397-supplementary.pdf]

Supplementary Information (SI)

Exploring DIX-DIX Homo- and Hetero-Oligomers in Wnt Signaling with AlphaFold2

Zehua Wen [1], Lei Wang [1], Shi-Wei Liu [1], Hua-Jun Shawn Fan [1], Jong-Won Song [2], And Ho-Jin Lee [3]

[1] College of Chemical Engineering, Sichuan University of Science and Engineering, Zigong City, Sichuan Province, P. R. China, 64300.

[2] Department of Chemistry Education, Daegu University, Daegudae-ro 201, Gyeongsan-si, Gyeongsangbuk-do, 38453, Republic of Korea.

[3] Division of Natural & Mathematical Sciences, LeMoyne-Owen College, Memphis, TN, 38126, USA

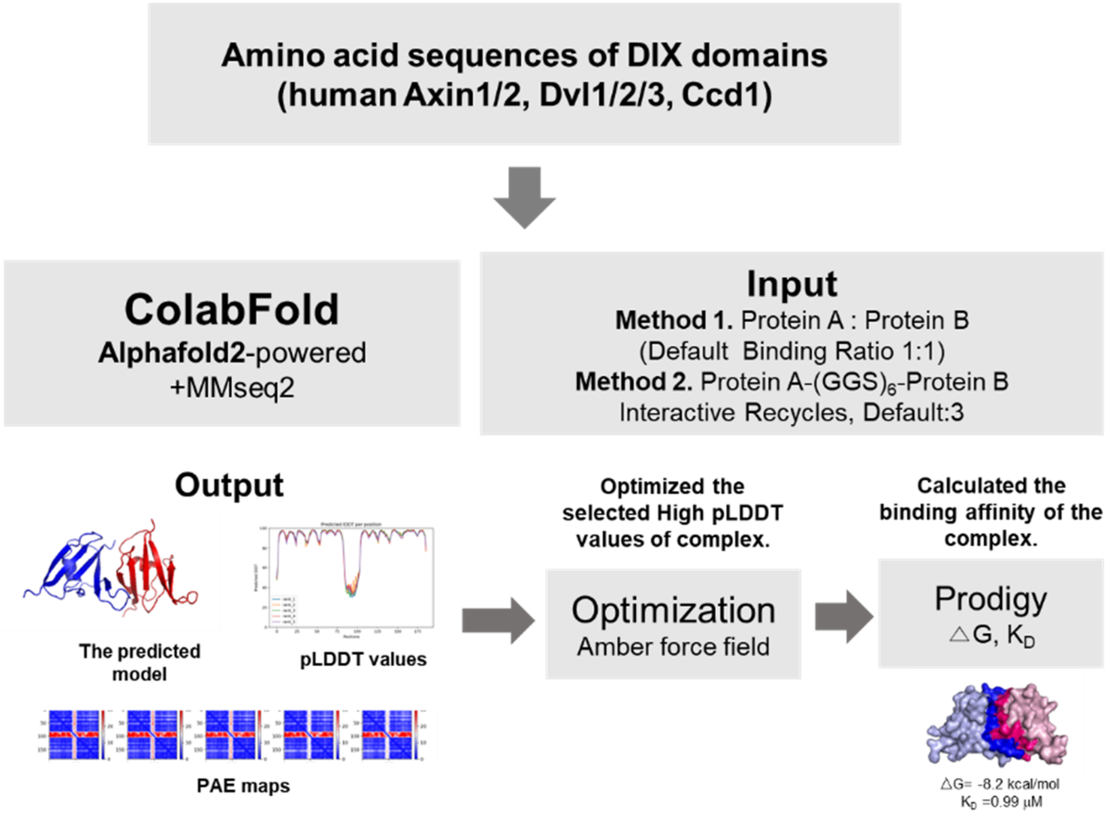

Figure S1. A flow chart for predicting homodimers and heterodimers of DIX domains. The amino acid sequences of DIX domains were obtained from UniProt. AlphaFold2-powered ColabFold was used (https://colab.research.google.com/github/sokrypton/ColabFold/blob/main/AlphaFold2.ipynb#scrollTo=kOblAo-xetgx). The outputs of ColabFold consist of three parts: (1) the predicted top five ranked 3D models; (2) the value of the predicted Local Distance Difference Test (pLDDT), which shows the prediction confidence for each residue in each model; (3) a matrix plot of predicted Aligned Error (PAE), which suggests the potential contacts between each of the two residues.

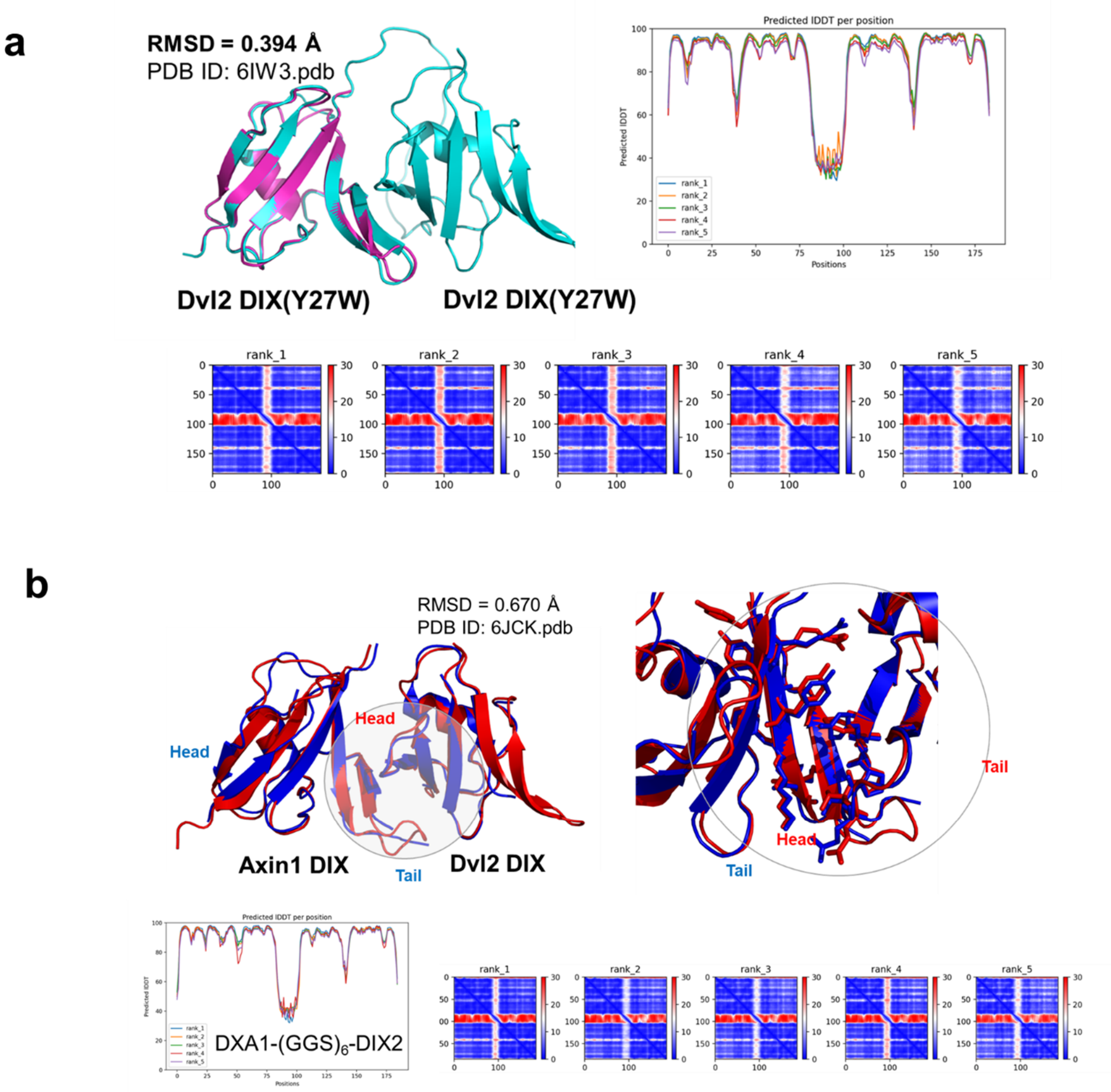

Figure S2. Evaluation of AF2 prediction. Excellent prediction of (a) Dvl2 DIX-M4(Y27W) homodimer and (b) Axin1 DIX and Dvl2 DIX heterodimer. An overlapped cartoon model of structures for the predicted and experimentally determined structures is shown. The pLDDT and PAE values are displayed. The high PAE value indicates the 6x GGS linker.

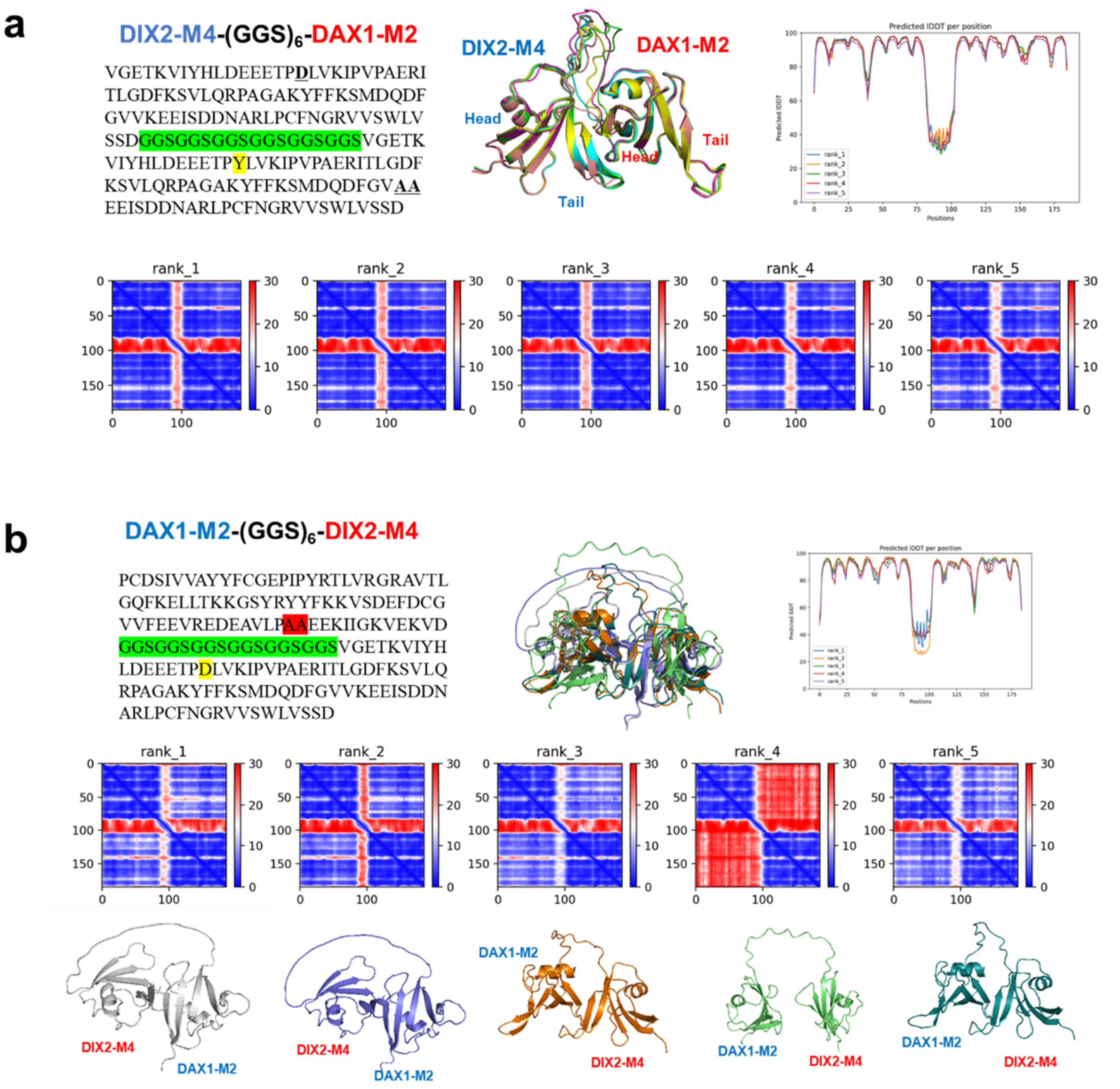

Figure S3. Evaluation of AF2 prediction with (a) DIX2-M4 ↔ DAX1-M2 and (b) DAX1-M2 ↔ DIX2-M4. Two constructs have a mutation in the head surface (M4) and the tail surface (M2). The sequence information of both proteins is given. The 6x(GGS) link is highlighted. The overlapped complex structures of both models are shown. The pLDDT and PAE values are shown.

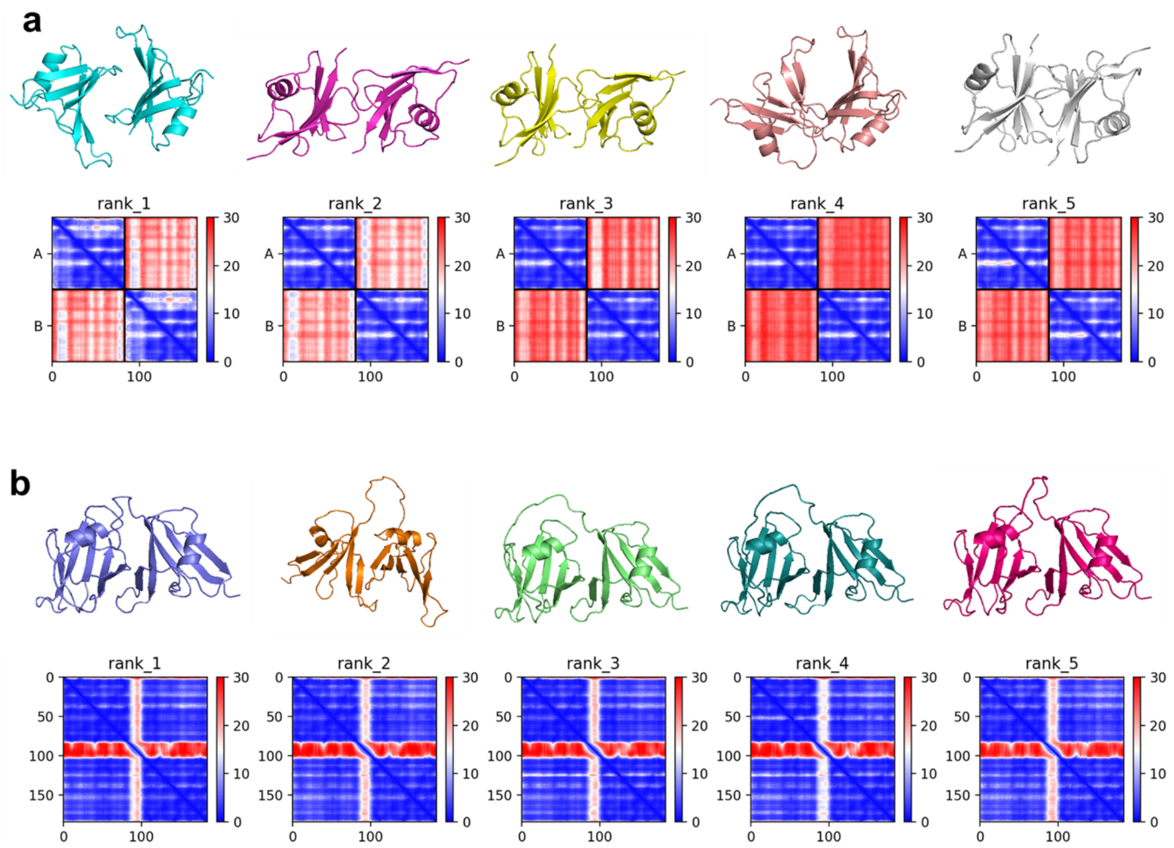

Figure S4. The AF2-powered complex structures prediction of Axin2 DIX (DAX2) homodimer. (a) The first method shows a high PAE (red indicates the unconfident), indicating a low confidence prediction. (b) The second method shows a low PAE value, supporting a high confident prediction. The high PAE values come from 6 x (GGS) linker residues. The head-to-tail structure was found for all top 5 complex structures for DAX2-$(GGS)_6$-DAX2.

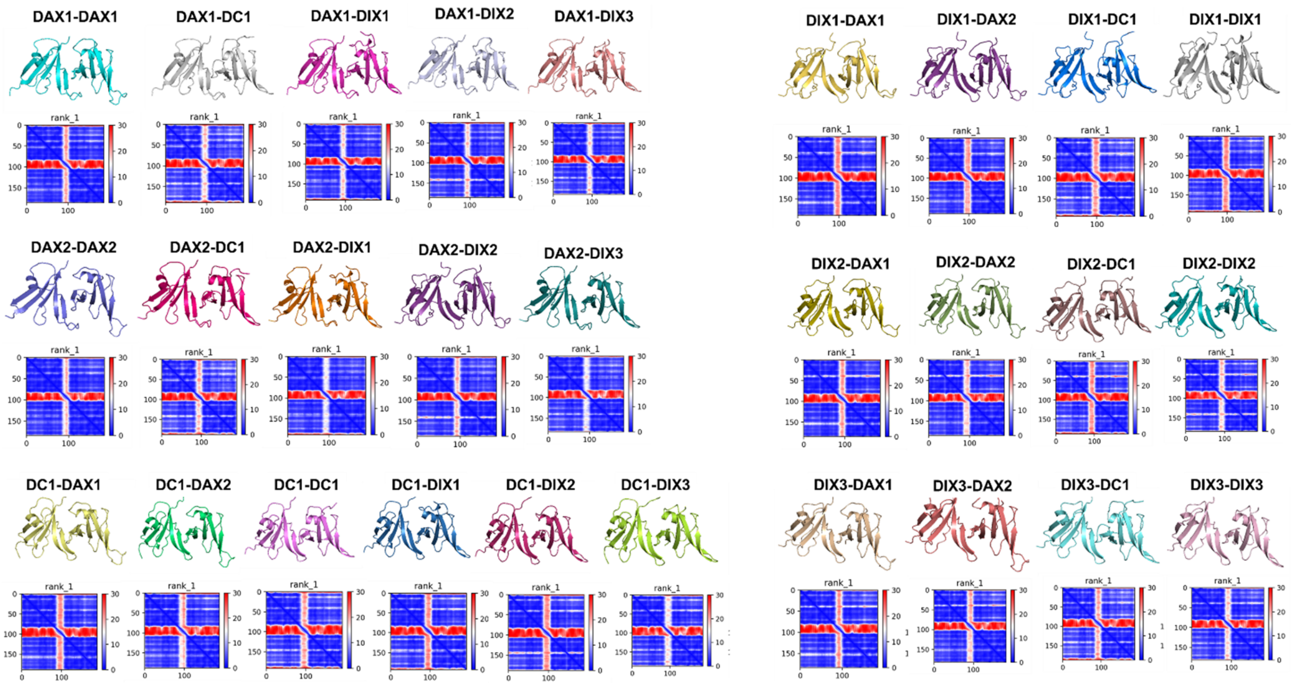

Figure S5. The AF2-predicted complex structures of homodimers and heterodimers are shown as illustration models (the same color of each complex used in Fig. 2a). Histogram plots of PAE value show the confidence level (blue to red indicates confidence to not confident) about the relative position of two regions in the protein complex. The top-ranked complex structure with a high pLDDT value and low PAE value was selected for further analysis.

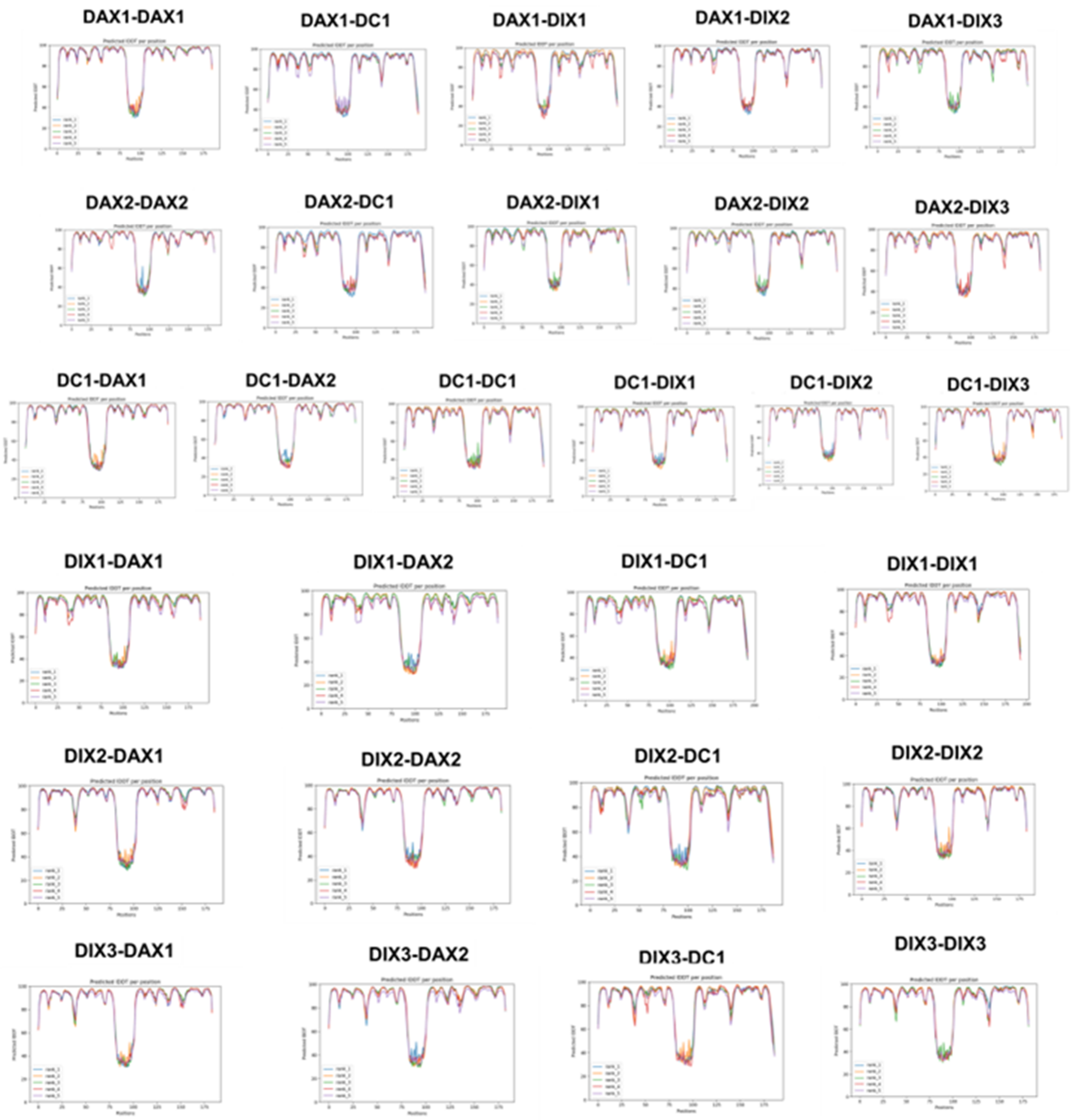

Figure S6. The values of pLDDT of top 5 ranked complex structures of the DIX domains.

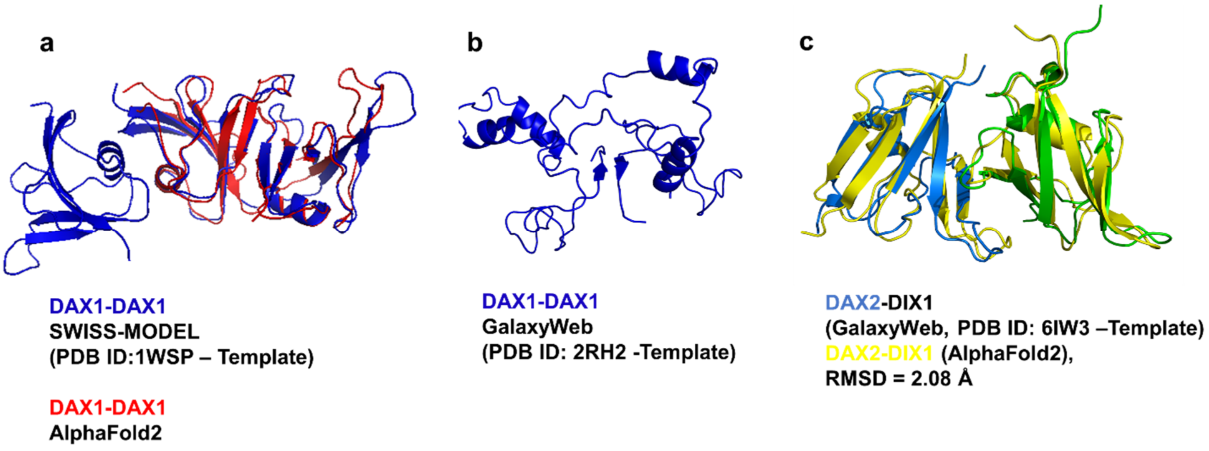

Figure S7. Homodimer and Heterodimer structures are predicted by (**a**) Templated-based homology modeling server, SWISS-MODEL, (**b**) Template-based homology modeling server GalaxyWeb, (**c**) Templated-based homology heterodimers GalaxyWeb server. The color represents the protein name. DAX1 is Axin1 DIX; DAX2 is Axin2 DIX; DIX1 is Dvl1 DIX domain.

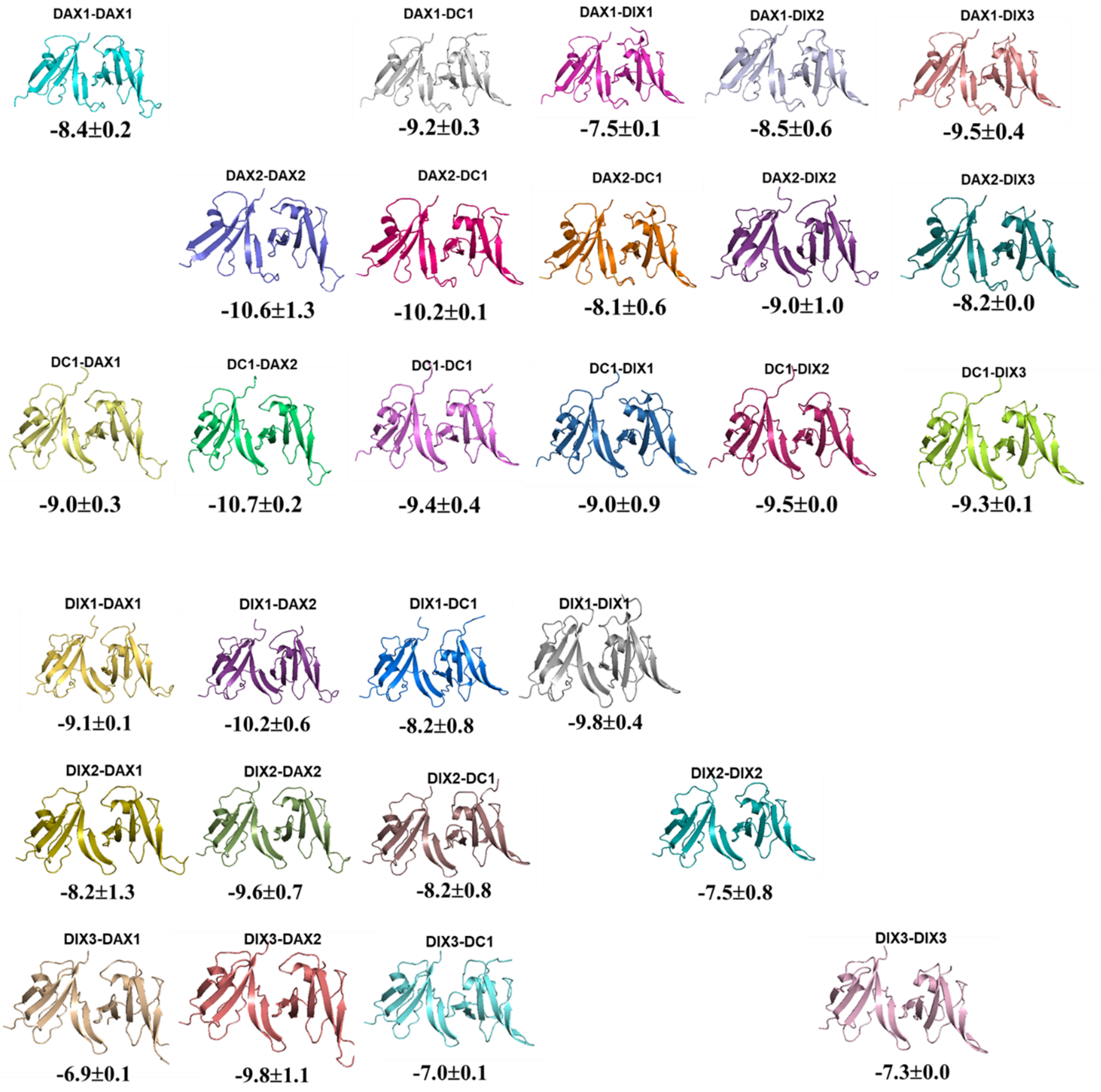

Figure S8. The binding affinities of complex structures were calculated using the PRODIGY web server. The colors of the illustrations represent the homodimers and heterodimers found in Fig. 2.

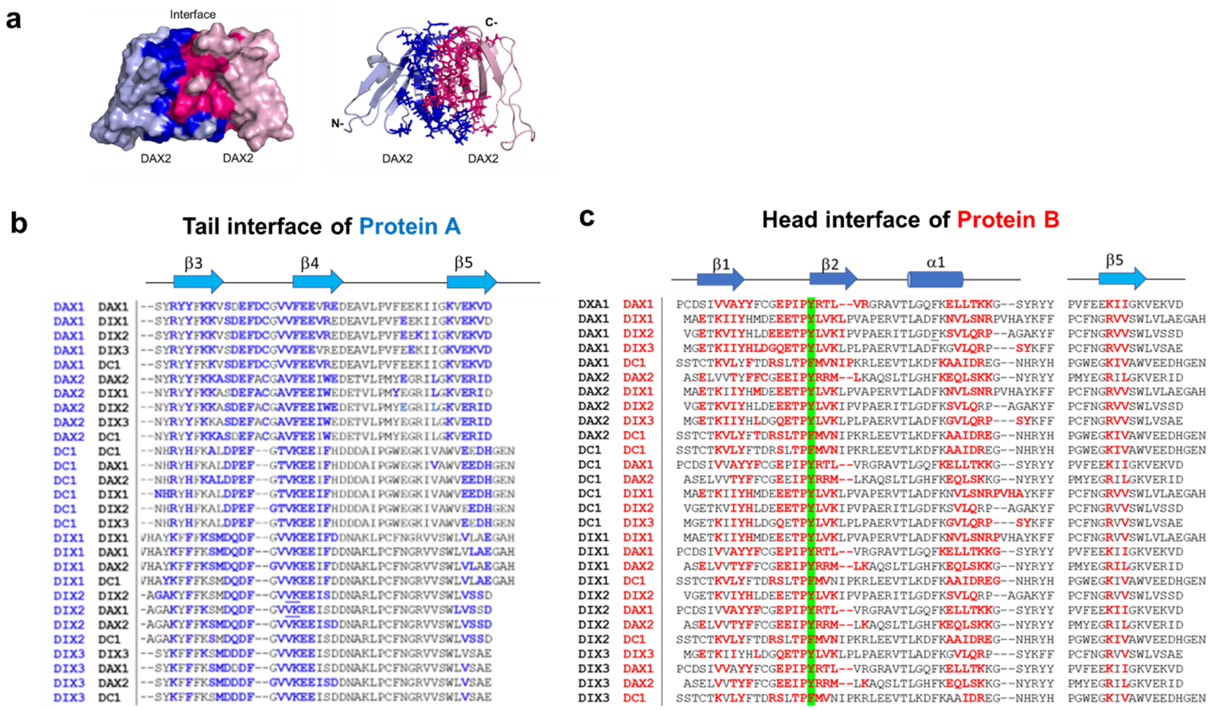

Figure S9. Residues involved in the homotypic and heterotypic protein-protein interaction are shown (**a**) The surface of the DAX2-DAX2 complex structure; cartoon structure of the homotypic DAX2-DAX2 complex. (**b** and **c**) The multiple sequence alignment of three DIX proteins and their paralogs are used to define the sequence regions of key secondary structure elements (**b**) Blue in color represents the tail interface of Protein A; (**c**) Red in color represents the head interface of Protein B from the AF2-predicted complex structure of DIX domains. The loop regions of the β3-strand and the β4-stand in the tail interface of Protein A; the α1-helix and the β3-strand in the head interface of Protein B may be critical to the binding specificity.

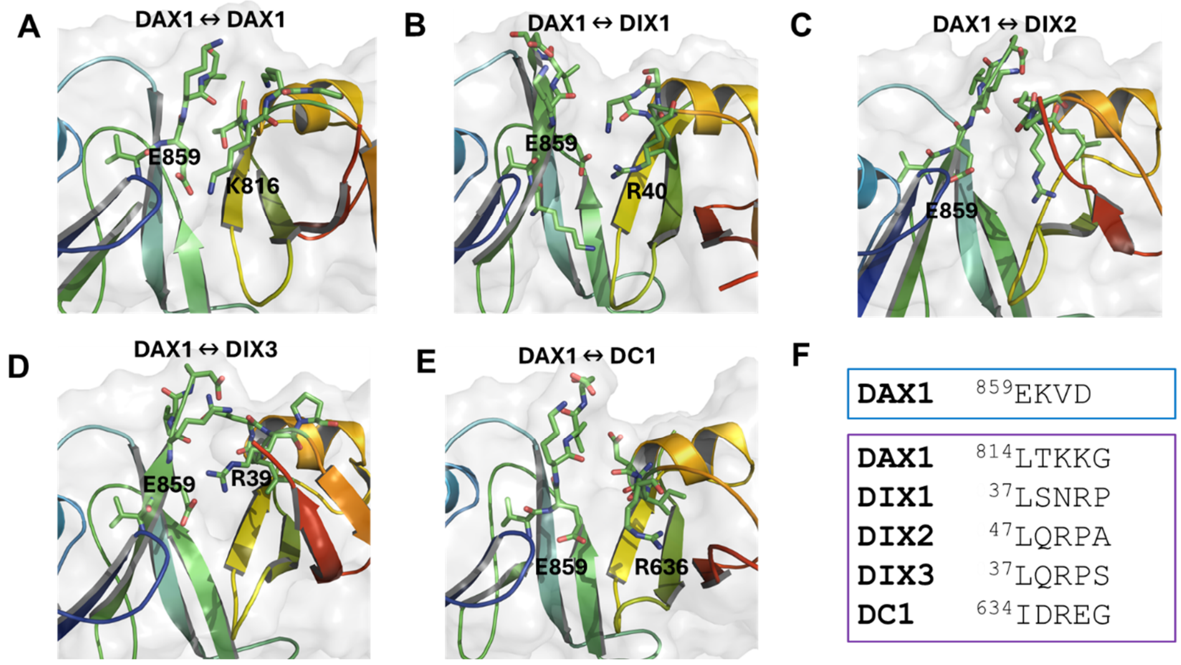

Figure S10. The interface of the tail region (the end of strand β5 of Protein A) and the head region (the C-terminus of α-helix of protein B) is shown. (**a**) DAX1-DAX1 (**b**) DAX1-DIX1 (**c**) DAX1-DIX2 (**d**) DAX1-DIX3 (**e**) DAX1-DC1. (**f**) the sequence information at the interface of Protein A(DAX1) and protein B (DAX1, DIX1/2/3, and DC1). (Sequence Information obtained from UniProt, human Axin1(ID: O15169-1), hDVL1(ID:O14640), hDVL2 (O14641), hDVL3(ID: Q92997), hDIXC1(ID:Q155Q3)

1.1 Supplementary Tables

Table S1. The reported X-ray structures of DIX proteins.

DIX Proteins	PDB ID	Method	Resolution
Rattus Axin1-DIX	1WSP	X-ray	2.90Å
Mouse Dvl1-DIX (Y17D)	3PZ8	X-ray	2.87Å
Human DVl2-DIX(Y27W/C80S)	6IW3	X-ray	1.64Å
Human Dvl2-DIX	4WIP	X-ray	2.69Å
Mouse Dvl2 DIX	6VCCC	Cryo-EM	3.60Å
Human Ccd1-DIX	3P27	X-ray	2.44Å
Mouse Ccd1 DIX	5Y3B	X-ray	3.00 Å
Axin1-DIX(Y760D) ↔ Dvl2 DIX(V67A/K68A),	6JCK	X-ray	3.09Å

Table S2. Input Sequence information of DIX domains from three proteins (Axin, Dvl, Ccd1) and their paralogs to predict the complex structures in this study using Alphafold2-powered ColabFold. The gray highlighted proteins are mutants. The underline below the letter indicates the mutated residue.

Protein A	Protein B	Sequences
DAX1 (A1)	DAX1(A1)	PCDSIVVAYYFCGEPIPYRTLVRGRAVTLGQFKELLTKKGSYRYYFKKVS<br>DEFDCGVVFEEVREDEAVLPVFEEKIIGKVEKVDGGSGGSGGSGGSGGS<br>GGSPCDSIVVAYYFCGEPIPYRTLVRGRAVTLGQFKELLTKKGSYRYYFK<br>KVSDEFDCGVVFEEVREDEAVLPVFEEKIIGKVEKVD
DAX2 (A2)	DAX2 (A2)	ASELVVTYFFCGEEIPYRRMLKAQSLTLGHFKEQLSKKGNYRYYFKKAS<br>DEFACGAVFEEIWEDETVLPMYEGRILGKVERIDGGSGGSGGSGGSGGS<br>GGSASELVVTYFFCGEEIPYRRMLKAQSLTLGHFKEQLSKKGNYRYYFK<br>KASDEFACGAVFEEIWEDETVLPMYEGRILGKVERID
DAX1-M3	DAX1-M2	PCDSIVVAYYFCGEPAPYATLVRGRAVTLGQFKELLTKKGSYRYYFKKV<br>SDEFDCGVVFEEVREDEAVLPVFEEKIIGKVEKVDGGSGGSGGSGGSGGS<br>GGSPCDSIVVAYYFCGEPIPYRTLVRGRAVTLGQFKELLTKKGSYRYYFK<br>KVSDEFDCGVVFEEVREDEAVLPAAEEKIIGKVEKVD
DC1 (C1)	DC1 (C1)	SSTCTKVLYFTDRSLTPFMVNIPKRLEEVTLKDFKAAIDREGNHRYHFKA<br>LDPEFGTVKEEIFHDDDAIPGWEGKIVAWVEEDHGENGGSGGSGGSGGS<br>GGSGGSSSTCTKVLYFTDRSLTPFMVNIPKRLEEVTLKDFKAAIDREGNH<br>RYHFKALDPEFGTVKEEIFHDDDAIPGWEGKIVAWVEEDHGEN
DIX1 (D1)	DIX1 (D1)	MAETKIIYHMDEEETPYLVKLPVAPERVTLADFKNVLSNRPVHAYKFFF<br>KSMDQDFGVVKEEIFDDNAKLPCFNGRVVSWLVLAEGAHGGSGGSGGS<br>GGSGGSGGSMAETKIIYHMDEEETPYLVKLPVAPERVTLADFKNVLSNR<br>PVHAYKFFFKSMDQDFGVVKEEIFDDNAKLPCFNGRVVSWLVLAEGAH
DIX2 (D2)	DIX2 (D2)	VGETKVIYHLDEEETPYLVKIPVPAERITLGDFKSVLQRPAGAKYFFKSM<br>DQDFGVVKEEISDDNARLPCFNGRVVSWLVSSDGGSGGSGGSGGSGGSG<br>GSVGETKVIYHLDEEETPYLVKIPVPAERITLGDFKSVLQRPAGAKYFFKS<br>MDQDFGVVKEEISDDNARLPCFNGRVVSWLVSSD
DIX2-M4<br>(D2-M4)<br>**Y27D**	DIX2-M2<br>(D2-M2)	VGETKVIYHLDEEETPDLVKIPVPAERITLGDFKSVLQRPAGAKYFFKSM<br>DQDFGVVKEEISDDNARLPCFNGRVVSWLVSSDGGSGGSGGSGGSGGSG<br>GSVGETKVIYHLDEEETPYLVKIPVPAERITLGDFKSVLQRPAGAKYFFKS<br>MDQDFGVAAEEISDDNARLPCFNGRVVSWLVSSD
DIX2-<br>M2(Y27W)	DIX2-<br>M2(Y27W)	VGETKVIYHLDEEETPWLVKIPVPAERITLGDFKSVLQRPAGAKYFFKSM<br>DQDFGVVKEEISDDNARLPCFNGRVVSWLVSSDGGSGGSGGSGGSGGSG<br>GSVGETKVIYHLDEEETPWLVKIPVPAERITLGDFKSVLQRPAGAKYFFK<br>SMDQDFGVVKEEISDDNARLPCFNGRVVSWLVSSD
DIX3 (D3)	DIX3 (D3)	MGETKIIYHLDGQETPYLVKLPLPAERVTLADFKGVLQRPSYKFFFKSMD<br>DDFGVVKEEISDDNAKLPCFNGRVVSWLVSAEGGSGGSGGSGGSGGSG<br>GSMGETKIIYHLDGQETPYLVKLPLPAERVTLADFKGVLQRPSYKFFFKS<br>MDDDFGVVKEEISDDNAKLPCFNGRVVSWLVSAE
DAX1 (A1)	DIX1 (D1)	PCDSIVVAYYFCGEPIPYRTLVRGRAVTLGQFKELLTKKGSYRYYFKKVS<br>DEFDCGVVFEEVREDEAVLPVFEEKIIGKVEKVDGGSGGSGGSGGSGGS<br>GGSMAETKIIYHMDEEETPYLVKLPVAPERVTLADFKNVLSNRPVHAYK<br>FFFKSMDQDFGVVKEEIFDDNAKLPCFNGRVVSWLVLAEGAH
DAX1 (A1)	DIX2 (D2)	PCDSIVVAYYFCGEPIPYRTLVRGRAVTLGQFKELLTKKGSYRYYFKKVS<br>DEFDCGVVFEEVREDEAVLPVFEEKIIGKVEKVDGGSGGSGGSGGSGGS<br>GGSVGETKVIYHLDEEETPYLVKIPVPAERITLGDFKSVLQRPAGAKYFF<br>KSMDQDFGVVKEEISDDNARLPCFNGRVVSWLVSSD
DAX1 (A1)	DIX3 (D3)	PCDSIVVAYYFCGEPIPYRTLVRGRAVTLGQFKELLTKKGSYRYYFKKVS<br>DEFDCGVVFEEVREDEAVLPVFEEKIIGKVEKVDGGSGGSGGSGGSGGS<br>GGSMGETKIIYHLDGQETPYLVKLPLPAERVTLADFKGVLQRPSYKFFFK<br>SMDDDFGVVKEEISDDNAKLPCFNGRVVSWLVSAE
DAX1-M3<br>(A1-M3)	DIX2-M2<br>(D2-M2)	PCDSIVVAYYFCGEPAPYATLVRGRAVTLGQFKELLTKKGSYRYYFKKV<br>SDEFDCGVVFEEVREDEAVLPVFEEKIIGKVEKVDGGSGGSGGSGGSGGS

		GGSVGETKVIYHLDEEETPYLVKIPVPAERITLGDFKSVLQRPAGAKYFF KSMDQDFGVAAEEISDDNARLPCFNGRVVSWLVSSD
DAX2 (A2)	DIX1 (D1)	ASELVVTYFFCGEEIPYRRMLKAQSLTLGHFKEQLSKKGNYRYYFKKAS DEFACGAVFEEIWEDETVLPMYEGRILGKVERIDGGSGGSGGSGGSGGS GGSMAETKIIYHMDEEETPYLVKLPVAPERVTLADFKNVLSNRPVHAYK FFFKSMDQDFGVVKEEIFDDNAKLPCFNGRVVSWLVLAEGAH
DAX2 (A2)	DIX2 (D2)	ASELVVTYFFCGEEIPYRRMLKAQSLTLGHFKEQLSKKGNYRYYFKKAS DEFACGAVFEEIWEDETVLPMYEGRILGKVERIDGGSGGSGGSGGSGGS GGSVGETKVIYHLDEEETPYLVKIPVPAERITLGDFKSVLQRPAGAKYFF KSMDQDFGVVKEEISDDNARLPCFNGRVVSWLVSSD
DAX2 (A2)	DIX3 (D3)	ASELVVTYFFCGEEIPYRRMLKAQSLTLGHFKEQLSKKGNYRYYFKKAS DEFACGAVFEEIWEDETVLPMYEGRILGKVERIDGGSGGSGGSGGSGGS GGSMGETKIIYHLDGQETPYLVKLPLPAERVTLADFKGVLQRPSYKFFFK SMDDDFGVVKEEISDDNAKLPCFNGRVVSWLVSAE
DIX1 (D1)	DAX1 (A1)	MAETKIIYHMDEEETPYLVKLPVAPERVTLADFKNVLSNRPVHAYKFFF KSMDQDFGVVKEEIFDDNAKLPCFNGRVVSWLVLAEGAHGGSGGSGGS GGSGGSGGSPCDSIVVAYYFCGEPIPYRTLVRGRAVTLGQFKELLTKKGS YRYYFKKVSDEFDCGVVFEEVREDEAVLPVFEEKIIGKVEKVD
DIX2 (D2)	DAX1 (A1)	VGETKVIYHLDEEETPYLVKIPVPAERITLGDFKSVLQRPAGAKYFFKSM DQDFGVVKEEISDDNARLPCFNGRVVSWLVSSDGGSGGSGGSGGSGGSG GSPCDSIVVAYYFCGEPIPYRTLVRGRAVTLGQFKELLTKKGSYRYYFKK VSDEFDCGVVFEEVREDEAVLPVFEEKIIGKVEKVD
DIX2 M4 (D2-M4)	DAX1 M2(A1-M2)	VGETKVIYHLDEEETPDLVKIPVPAERITLGDFKSVLQRPAGAKYFFKSM DQDFGVVKEEISDDNARLPCFNGRVVSWLVSSDGGSGGSGGSGGSGGSG GSPCDSIVVAYYFCGEPIPYRTLVRGRAVTLGQFKELLTKKGSYRYYFKK VSDEFDCGVVFEEVREDEAVLPAAEEKIIGKVEKVD
DAX1 M2(A1-M2) (Negative Control)	DIX2 M4 (D2-M4) (See Fig. S5).	PCDSIVVAYYFCGEPIPYRTLVRGRAVTLGQFKELLTKKGSYRYYFKKVS DEFDCGVVFEEVREDEAVLPAAEEKIIGKVEKVDGGSGGSGGSGGSGGS GGSVGETKVIYHLDEEETPDLVKIPVPAERITLGDFKSVLQRPAGAKYFF KSMDQDFGVVKEEISDDNARLPCFNGRVVSWLVSSD
DIX3 (D3)	DAX1 (A1)	MGETKIIYHLDGQETPYLVKLPLPAERVTLADFKGVLQRPSYKFFFKSMD DDFGVVKEEISDDNAKLPCFNGRVVSWLVSAEGGSGGSGGSGGSGGSG GSPCDSIVVAYYFCGEPIPYRTLVRGRAVTLGQFKELLTKKGSYRYYFKK VSDEFDCGVVFEEVREDEAVLPVFEEKIIGKVEKVD
DIX1 (D1)	DAX2 (A2)	MAETKIIYHMDEEETPYLVKLPVAPERVTLADFKNVLSNRPVHAYKFFF KSMDQDFGVVKEEIFDDNAKLPCFNGRVVSWLVLAEGAHGGSGGSGGS GGSGGSGGSASELVVTYFFCGEEIPYRRMLKAQSLTLGHFKEQLSKKGN YRYYFKKASDEFACGAVFEEIWEDETVLPMYEGRILGKVERID
DIX2 (D2)	DAX2 (A2)	VGETKVIYHLDEEETPYLVKIPVPAERITLGDFKSVLQRPAGAKYFFKSM DQDFGVVKEEISDDNARLPCFNGRVVSWLVSSDGGSGGSGGSGGSGGSG GSASELVVTYFFCGEEIPYRRMLKAQSLTLGHFKEQLSKKGNYRYYFKK ASDEFACGAVFEEIWEDETVLPMYEGRILGKVERID
DIX3 (D3)	DAX2 (A2)	MGETKIIYHLDGQETPYLVKLPLPAERVTLADFKGVLQRPSYKFFFKSMD DDFGVVKEEISDDNAKLPCFNGRVVSWLVSAEGGSGGSGGSGGSGGSG GSASELVVTYFFCGEEIPYRRMLKAQSLTLGHFKEQLSKKGNYRYYFKK ASDEFACGAVFEEIWEDETVLPMYEGRILGKVERID
DAX1 (A1)	DC1 (C1)	PCDSIVVAYYFCGEPIPYRTLVRGRAVTLGQFKELLTKKGSYRYYFKKVS DEFDCGVVFEEVREDEAVLPVFEEKIIGKVEKVDGGSGGSGGSGGSGGS GGSSSTCTKVLYFTDRSLTPFMVNIPKRLEEVTLKDFKAAIDREGNHRYH FKALDPEFGTVKEEIFHDDDAIPGWEGKIVAWVEEDHGEN
DAX2 (A2)	DC1 (C1)	ASELVVTYFFCGEEIPYRRMLKAQSLTLGHFKEQLSKKGNYRYYFKKAS DEFACGAVFEEIWEDETVLPMYEGRILGKVERIDGGSGGSGGSGGSGGS GGSSSTCTKVLYFTDRSLTPFMVNIPKRLEEVTLKDFKAAIDREGNHRYH FKALDPEFGTVKEEIFHDDDAIPGWEGKIVAWVEEDHGEN
DC1 (C1)	DAX1 (A1)	SSTCTKVLYFTDRSLTPFMVNIPKRLEEVTLKDFKAAIDREGNHRYHFKA LDPEFGTVKEEIFHDDDAIPGWEGKIVAWVEEDHGENGGSGGSGGSGGS

		GGSGGSPCDSIVVAYYFCGEPIPYRTLVRGRAVTLGQFKELLTKKGSYRY<br>YFKKVSDEFDCGVVFEEVREDEAVLPVFEEKIIGKVEKVD
DC1 (C1)	DAX2 (A2)	SSTCTKVLYFTDRSLTPFMVNIPKRLEEVTLKDFKAAIDREGNHRYHFKA<br>LDPEFGTVKEEIFHDDDAIPGWEGKIVAWVEEDHGENGGSGGSGGSGGS<br>GGSGGSASELVVTYFFCGEEIPYRRMLKAQSLTLGHFKEQLSKKGNYRY<br>YFKKASDEFACGAVFEEIWEDETVLPMYEGRILGKVERID
DIX1 (D1)	DC1 (C1)	MAETKIIYHMDEEETPYLVKLPVAPERVTLADFKNVLSNRPVHAYKFFF<br>KSMDQDFGVVKEEIFDDNAKLPCFNGRVVSWLVLAEGAHGGSGGSGGS<br>GGSGGSGGSSSTCTKVLYFTDRSLTPFMVNIPKRLEEVTLKDFKAAIDRE<br>GNHRYHFKALDPEFGTVKEEIFHDDDAIPGWEGKIVAWVEEDHGEN
DIX2 (D2)	DC1 (C1)	VGETKVIYHLDEEETPYLVKIPVPAERITLGDFKSVLQRPAGAKYFFKSM<br>DQDFGVVKEEISDDNARLPCFNGRVVSWLVSSDGGSGGSGGSGGSGGSG<br>GSSSTCTKVLYFTDRSLTPFMVNIPKRLEEVTLKDFKAAIDREGNHRYHF<br>KALDPEFGTVKEEIFHDDDAIPGWEGKIVAWVEEDHGEN
DIX3 (D3)	DC1 (C1)	MGETKIIYHLDGQETPYLVKLPLPAERVTLADFKGVLQRPSYKFFFKSMD<br>DDFGVVKEEISDDNAKLPCFNGRVVSWLVSAEGGSGGSGGSGGSGGSG<br>GSSSTCTKVLYFTDRSLTPFMVNIPKRLEEVTLKDFKAAIDREGNHRYHF<br>KALDPEFGTVKEEIFHDDDAIPGWEGKIVAWVEEDHGEN
DC1 (C1)	DIX1 (D1)	SSTCTKVLYFTDRSLTPFMVNIPKRLEEVTLKDFKAAIDREGNHRYHFKA<br>LDPEFGTVKEEIFHDDDAIPGWEGKIVAWVEEDHGENGGSGGSGGSGGS<br>GGSGGSMAETKIIYHMDEEETPYLVKLPVAPERVTLADFKNVLSNRPVH<br>AYKFFFKSMDQDFGVVKEEIFDDNAKLPCFNGRVVSWLVLAEGAH
DC1 (C1)	DIX2 (D2)	SSTCTKVLYFTDRSLTPFMVNIPKRLEEVTLKDFKAAIDREGNHRYHFKA<br>LDPEFGTVKEEIFHDDDAIPGWEGKIVAWVEEDHGENGGSGGSGGSGGS<br>GGSGGSVGETKVIYHLDEEETPYLVKIPVPAERITLGDFKSVLQRPAGAK<br>YFFKSMDQDFGVVKEEISDDNARLPCFNGRVVSWLVSSD
DC1 (C1)	DIX3 (D3)	SSTCTKVLYFTDRSLTPFMVNIPKRLEEVTLKDFKAAIDREGNHRYHFKA<br>LDPEFGTVKEEIFHDDDAIPGWEGKIVAWVEEDHGENGGSGGSGGSGGS<br>GGSGGSMGETKIIYHLDGQETPYLVKLPLPAERVTLADFKGVLQRPSYKF<br>FFKSMDDDFGVVKEEISDDNAKLPCFNGRVVSWLVSAE

Table S3. Thermodynamic parameters (△G, in kcal/mol; K_D in μM) of homodimers and heterodimers of DIX domains obtained by the PRODIGY server. The AlphaFold2-predicted complex structures obtained by two methods described in the method section were optimized using the Amber force field. [a]

Molecule A	Molecule B	^{a}△G	$^a K_D$	^{b}△G	$^b K_D$
DAX1	DAX1	-8.5	0.56	-8.2	0.99
DAX1-M3	DAX1-M2	-8.1	1.2	-8.1	1.2
DAX2	DAX2	-9.6	0.096	-11.5	0.0035
DC1	DC1	-9.6	0.084	-9.1	0.20
DIX1	DIX1	-10.0	0.047	-9.5	0.12
DIX2	DIX2	-6.9	8.0	-8.0	1.3
DIX2-M4	DIX2-M2	-7.3	4.7	-7.3	4.7
DIX3	DIX3	-7.3	4.8	-7.3	4.8
DAX1	DIX1	-7.4	3.6	-7.6	2.7
DAX1	DIX2	-8.0	1.3	-8.9	0.31
DAX1-M3	DIX2-M2	-8.6	0.50	-7.6	2.5
DAX1	DIX3	-9.8	0.070	-9.2	0.19
DIX1	DAX1	-9.0	0.24	-9.1	0.22
DIX2	DAX1	-9.1	0.21	-7.2	4.8
DIX2-M4	DAX1-M2	-6.8	9.8	-6.4	19
DIX3	DAX1	-6.8	9.6	-6.9	9.0
DAX2	DIX1	-7.6	2.9	-8.5	0.56
DAX2	DIX2	-9.7	0.080	-8.3	0.81
DAX2	DIX3	-8.2	0.93	-8.2	0.93
DIX1	DAX2	-10.6	0.016	-9.7	0.083
DIX2	DAX2	-9.1	0.21	-10.1	0.039
DIX3	DAX2	-9.8	0.060	-9.8	0.060
DIX1	DC1	-7.4	3.9	-9.0	0.27
DIX2	DC1	-8.7	0.45	-7.6	2.8
DIX3	DC1	-7.1	6.6	-6.9	8.0
DC1	DIX1	-8.3	0.83	-9.6	0.084
DC1	DIX2	-9.5	0.10	-9.5	0.10
DC1	DIX3	-9.3	0.14	-9.2	0.17
DAX1	DC1	-9.4	0.14	-9.0	0.27
DC1	DAX1	-9.3	0.14	-8.6	0.50
DAX2	DC1	-10.1	0.039	-10.3	0.027
DC1	DAX2	-10.5	0.020	-10.8	0.012

[a.] The Alphafold2-predicted Complex structure of DIX domains obtained from (a) Method 1 and (b) Method 2. The K_D value was calculated from △G=-RT ln K_D, T=298 K, R=8.314 J/mol K.

Table S4. The binding energies (△G, in kcal/mol; K_D in μM) of homodimers and heterodimers of DIX domains were obtained by the HawkDock.[a]

Protein A	Protein B	△G	△△G	Protein A	Protein B	△G	△△G
DAX1	DAX1	-116.13	0.00	DAX2	DAX2	-119.48	-3.35
DC1	DC1	[b] N/A	-	DIX1	DIX1	-111.17	4.96
DIX2	DIX2	-99.38	16.75	DIX3	DIX3	-96.88	19.25
DAX1	DIX1	-102.36	13.77	DIX1	DAX1	-111.61	4.52
DAX1	DIX2	-109.38	6.75	DIX2	DAX1	-80.61	35.52
DAX1	DIX3	-125.52	-9.39	DIX3	DAX1	[b] N/A	-
DAX1	DC1	-82.28	33.85	DC1	DAX1	-65.53	50.6
DAX2	DIX1	-54.92	61.21	DIX1	DAX2	-132.1	-15.97
DAX2	DIX2	-93.05	23.08	DIX2	DAX2	-127.93	-11.8
DAX2	DIX3	-119.06	-2.93	DIX3	DAX2	-119.23	-3.1
DAX2	DC1	-104.47	11.66	DC1	DAX2	-135.78	-19.65
DC1	DIX1	-122.47	-6.34	DIX1	DC1	-121.30	-5.17
DC1	DIX2	-120.20	-4.07	DIX2	DC1	-77.07	39.06
DC1	DIX3	-87.99	28.14	DIX3	DC1	[b] N/A	-

[a].MM/GBSA Analysis [b].n/a: no head-to-tail interaction was predicted.

Table S5. Analysis of Interfaces for DIX-mediated interactions.

Complexes	Number of Residues	Solvent-accessible area, Å	Solvation energy, kcal/mol		
Protein A/ Protein B	Interface (A/B)	Interface, Protein A/ Protein B	Isolated structure (A/B)	Gain on complex formation(A/B)	Average gain(A/B)
DAX1-DAX1	22/23	933.5 / 923.4	-64.2/-64.1	-0.8/-1.9	-6.2/-5.6
DAX1-DIX1	20/22	839.1 / 823.9	-64.8/-77.5	+0.6/-0.5	-4.9/-4.5
DAX1-DIX2	28/25	944.6 / 1005.1	-62.2/-63.7	-0.6/-1.3	-7.5/-5.0
DAX1-DIX3	24/29	998.0 / 1012.6	-61.0/-17.3	-1.1/+1.7	-7.6/-4.7
DAX1-DC1	22/23	868.2 / 827.1	-63.3/-62.7	-4.5/-2.3	-5.8/-4.2
DAX2-DAX2	27/24	980.5 / 1024.9	-68.7/-72.4	-1.9/1.3	-5.1/-4.0
DAX2-DIX1	21/23	812.5 / 816.8	-68.1/-72.7	-2.6/-1.6	-4.0/-5.7
DAX2-DIX2	24/17	745.6 / 747.3	-64.5/-64.3	-1.5/0.1	-5.0/-4.3
DAX2-DIX3	20/23	981.6 / 906.6	-69.1/-68.9	-1.9/-1.5	-4.3/-5.3
DAX2-DC1	24/25	889.1 / 939.9	-64.5/-59.7	-5.2/-2.8	-5.8/-5.8
DIX1-DIX1	21/24	950.8 / 879.1	-77.8/-70.2	0.2/-1.8	-5.1/-7.3
DIX1-DAX1	24/25	906.8 / 935.0	-72.0/-66.9	-3.5/2.6	-6.6/-5.3
DIX1-DAX2	29/26	1025.6 / 984.9	-74.4/-66.8	-1.6/-2.6	-6.5/-5.4
DIX1-DC1	20/25	788.3 / 775.8	-72.6/-61.4	-4.6/0.2	-5.6/-4.3
DIX2-DIX2	21/21	842.0 / 858.5	-64.2/-61.7	0.4/0.0	-4.6/-4.9
DIX2-DAX1	19/23	764.3 / 784.0	-65.3/-62.8	-1.2/-1.5	-4.2/-4.9
DIX2-DAX2	24/24	942.9 / 936.5	-62.9/-70.2	-0.2/2.2	-5.5/-4.1
DIX2-DC1	17/20	689.5 / 714.4	-62.4/-64.8	-3.1/1.0	-4.6/-3.0
DIX3-DIX3	18/19	687.9 / 632.6	-68.5/-66.4	-0.1/0.4	-4.2/-4.6
DIX3-DAX1	16/20	647.5 / 637.9	-68.4/-64.9	1.0/-1.8	-4.0/-3.8
DIX3-DAX2	25/21	839.3 / 794.5	-73.6/-70.1	-0.1/1.4	-4.3/-3.4
DIX3-DC1	16/16	603.5 / 608.4	-63.8/-60.1	-2.1/-0.9	-4.3/-3.2
DC1-DC1	23/23	811.5 / 804.1	-61.5/-62.6	0.0/0.3	-5.0/-4.7
DC1-DAX1	23/21	810.3 / 834.3	-59.3/-62.8	-2.6/-4.9	-5.1/-5.7
DC1-DAX2	25/26	948.3 / 954.6	-63.4/-67.0	-3.0/1.4	-5.1/-5.3
DC1-DIX1	25/28	1058.8 / 962.5	-62.9/-74.7	0.0/-4.5	-5.8/-6.3
DC1-DIX2	24/26	895.0 / 893.4	-62.6/-67.6	0.3/2.4	-4.5/-4.2
DC1-DIX3	23/27	907.0 / 796.3	-66.4/-71.2	1.8/1.5	-4.0/-4.7

Table S6. Hydrogen bonds and Salt bridges are found at the interface between the complex's tail (Protein A, after strand β5) and head(Protein B, between helix α1 and strand β3) areas in the interfaces of DIX-mediated Interaction.[a]

Protein A	Protein B	Dist.	Protein A	Protein B	Dist.	Protein A	Protein B	Dist.
**DAX1**	**DAX1**	Å	**DAX2**	**DAX2**	Å	**DC1**	**DC1**	Å
K857 [NZ]	E792 [OE1]	2.77	D843 [O]	K798 [NZ]	2.77	E677 [OE2]	R636 [NH1]	2.90
K857 [NZ]	E792 [OE2]	2.74	K838 [NZ]	E773 [OE1]	2.74	H680 [ND1]	E637 [OE2]	2.55
E859 [OE1]	K816 [NZ]	2.83	K838 [NZ]	E773 [OE2]	2.99	H680 [NE2]	E609 [OE2]	2.84
E859 [OE2]	K816 [NZ]	3.15				H680 [NE2]	E637 [OE2]	3.80
						E677 [OE2]	R636 [NE]	3.11
						E677 [OE2]	R636 [NH2]	2.90
**DAX1**	**DIX1**	Å	**DAX2**	**DIX1**	Å	**DC1**	**DIX1**	Å
K857 [NZ]	E14 [OE1]	2.72	K838 [NZ]	E14[OE1]	2.71	E677 [OE2]	R40[NE]	3.16
K857 [NZ]	T16 [O]	2.95	K838 [NZ]	E14[OE2]	2.83	E677 [OE2]	R40[NH2]	2.93
E859 [OE1]	N39 [ND2]	2.88	E839 [O]	N39[ND2]	3.70	D679 [OD2]	N39[DN2]	2.91
E859 [OE2]	R40 [NH1]	2.78	E839 [OE2]	R40[NH1]	2.81	E682 [OE1]	H43[NE2]	2.56
E859 [OE1]	R40 [NH2]	3.03	E839 [OE2]	R40[NH2]	2.90	E682 [OE2]	H43[NE2]	2.93
E859 [OE2]	R40 [NH1]	3.97				N683 [OD1]	N44[ND2]	2.91
E859 [OE2]	R40 [NH2]	2.99						
**DAX1**	**DIX2**	Å	**DAX2**	**DIX2**	Å	**DC1**	**DIX2**	Å
K857 [NZ]	E13 [OE1]	2.65	K838 [NZ]	E13[OE1]	2.70	D679 [OD2]	Q48[NE2]	2.85
K857 [NZ]	E13 [OE2]	3.45	K838 [NZ]	E13[OE2]	3.05	E682 [OE2]	Q48[NE2]	2.92
K857 [NZ]	T15 [O]	3.22	K838 [NZ]	T15[O]	3.14	E677 [OE1]	R49[NH1]	2.87
K860[NZ]	R49 [O]	2.92				E677 [OE2]	R49[NH2]	2.87
**DAX1**	**DIX3**	Å	**DAX2**	**DIX3**	Å	**DC1**	**DIX3**	Å
K857 [NZ]	Q13 [OE1]	2.80	K838 [NZ]	E15[OE1]	2.94	D679 [OD2]	Q38[NE2]	2.91
K857 [NZ]	Q13 [OE2]	2.93	K838 [NZ]	E15[OE2]	2.94	H680 [O]	R39[NH2]	2.89
K857 [NZ]	T15 [O]	2.83	K838 [NZ]	Y17[OH]	3.09	D679 [OD2]	Q38[NE2]	2.91
K860 [NZ]	E14 [OE2]	3.01	E839 [OE2]	R39[NH1]	2.90	H680 [O]	R39[NH2]	2.89
K860 [NZ]	E14 [OE1]	2.93	E839 [OE2]	R39[NH2]	2.87	N683 [O]	Q38[NE2]	2.94
E859 [OE2]	R39 [NH1]	2.86						
E859 [OE2]	R39 [NH2]	2.83						
**DAX1**	**DC1**	Å	**DAX2**	**DC1**	Å			
E859 [OE2]	R609 [NH2]	2.90	K838 [NZ]	R609 [O]	2.72			
E859 [OE2]	R609 [NE]	2.96	E839 [OE1]	R636 [NH2]	2.92			
			E839 [OE1]	R636 [NE]	2.92			
			R841 [NH1]	E637 [OE1]	2.85			
			R841 [NE]	E637 [OE1]	3.98			
**D1X1**	**DIX1**	Å	**DIX2**	**DIX2**	Å	D1X3	DIX3	N/A[a]
E85[OE1]	H43[NE2]	2.64	V80[O]	Q48 [NE2]	2.87			
E85[OE2]	H43[NH2]	3.49	S81[O]	Q48 [NE2]	2.87			
**D1X1**	**DAX1**	Å	**DIX2**	**DAX1**	N/A[b]	DIX3	DAX1	N/A[a]
E85[N]	E812[OE1]	3.48	DC1	**DAX1**	Å	**DC1**	**DAX2**	Å
E85[OE1]	K816[NZ]	2.95	E81[OE1]	K816 [NZ]	2.85	E682 [N]	S796 [N]	3.63
E85[OE2]	K816[NZ]	3.79	E81[OE2]	K816 [NZ]	3.95	D683 [OD1]	K798 [NZ]	2.78
L83[O]	K817[NZ]	3.08	E86[OE1]	K816 [NZ]	2.79			
E85[OE1]	K860[NZ]	2.86	E86[OE2]	K816 [NZ]	2.72			
E85[OE2]	K860[NZ]	2.78						
H88[NE2]	D862[OD2]	3.70						
**D1X1**	**DAX2**	Å	**DIX2**	**DAX2**	Å	**D1X3**	**DAX2**	Å
E85[OE2]	K798 [N]	3.54	S82[O]	K798 [NZ]	2.93	E82[O]	K797[NZ]	2.96
E85[OE1]	K798 [NZ]	2.82	D83[O]	K798 [NZ]	2.85			
E85[OE2]	K798 [NZ]	2.77						
**D1X1**	**DC1**	Å	DIX2	DC1	N/A[a]	DIX3	DC1	N/A[a]
E85[O]	R609 [NH2]	2.92						
E85[OE1]	R636 [NH2]	2.79						
E85[OE1]	R609 [NH2]	2.87						
E85[OE2]	R609 [NH1]	2.88						

[a.] The information on the hydrogen bond and salt bridge found at the interface of DIX-mediated interactions are deposited in Figshare: https://doi.org/10.6084/m9.figshare.27042208.

[b.] No hydrogen bond nor salt bridge was found.
